# *SNORA37*/CMTR1/ELAVL1 feedback loop drives gastric cancer progression via facilitating *CD44* alternative splicing

**DOI:** 10.1186/s13046-025-03278-x

**Published:** 2025-01-16

**Authors:** Banghe Bao, Minxiu Tian, Xiaojing Wang, Chunhui Yang, Jiaying Qu, Shunchen Zhou, Yang Cheng, Qiangsong Tong, Liduan Zheng

**Affiliations:** 1 Department of Pathology, Union Hospital, Tongji Medical College, Huazhong University of Science and Technology, 1277 Jiefang Avenue, Wuhan, 430022 Hubei Province People’s Republic of China; 2 Department of Pediatric Surgery, Union Hospital, Tongji Medical College, Huazhong University of Science and Technology, 1277 Jiefang Avenue, Wuhan, 430022 Hubei Province People’s Republic of China; 3 Department of Geriatrics, Union Hospital, Tongji Medical College, Huazhong University of Science and Technology, 1277 Jiefang Avenue, Wuhan, 430022 Hubei Province People’s Republic of China

**Keywords:** Small nucleolar RNA, Cap methyltransferase 1, ELAV like RNA binding protein 1, Alternative splicing, Cancer progression

## Abstract

**Background:**

Emerging evidence shows that small nucleolar RNA (snoRNA), a type of highly conserved non-coding RNA, is involved in tumorigenesis and aggressiveness. However, the roles of snoRNAs in regulating alternative splicing crucial for cancer progression remain elusive.

**Methods:**

High-throughput RNA sequencing and comprehensive analysis were performed to identify crucial snoRNAs and downstream alternative splicing events. Biotin-labeled RNA pull-down, mass spectrometry, cross-linking RNA immunoprecipitation, and in vitro binding assays were applied to explore interaction of snoRNAs with protein partners. Alternative splicing and gene expression was observed by real-time quantitative RT-PCR and western blot assays. In vitro and in vivo studies were performed to investigate biological effects of snoRNAs and their protein partners in gastric cancer. Survival analysis was undertaken by using Kaplan-Meier method and log-rank test.

**Results:**

*SNORA37* was identified as an up-regulated snoRNA essential for tumorigenesis and aggressiveness of gastric cancer. Gain- and loss-of-function studies indicated that *SNORA37* promoted the growth, invasion, and metastasis of gastric cancer cells in vitro and in vivo. Mechanistically, as an ELAV like RNA binding protein 1 (ELAVL1)-generated snoRNA, *SNORA37* directly bound to cap methyltransferase 1 (CMTR1) to facilitate its interaction with ELAVL1, resulting in nuclear retention and activity of ELAVL1 in regulating alternative splicing of *CD44*. Rescue studies revealed that *SNORA37* exerted oncogenic roles in gastric cancer progression via facilitating CMTR1-ELAVL1 interaction. In clinical gastric cancer cases, high levels of *SNORA37*, *CMTR1*, *ELAVL1*, or *CD44* were associated with shorter survival and poor outcomes of patients.

**Conclusions:**

These results indicated that *SNORA37*/CMTR1/ELAVL1 feedback loop drives gastric cancer progression via facilitating *CD44* alternative splicing.

**Supplementary Information:**

The online version contains supplementary material available at 10.1186/s13046-025-03278-x.

## Background

Gastric cancer is a prevalent and lethal malignancy around the world, and ranks fifth or fourth in terms of incidence or mortality rate, respectively [1]. Although recent advances in multiple therapeutic modalities, the outcomes of advanced gastric cancer patients remain elusive, mainly due to tumor recurrence or aggressiveness [2]. Therefore, it is urgent to explore mechanisms underlying gastric cancer progression for improving therapeutic efficiency. Alternative splicing, a process of removing introns from precursor messenger RNA (pre-mRNA) and combining various exons, is essential for mRNA complexity and protein diversity [3] via several patterns, including skipping exon (SE), mutually exclusive exons (MXE), alternative 3' splice sites (A3SS), alternative 5' splice sites (A5SS), and retained intron (RI) [4–6]. Previous studies show that alternative splicing is governed by spliceosomes, such as serine/arginine-rich (SR) proteins, heterogeneous nuclear ribonucleoprotein (HNRNP), and ELAV like RNA binding protein 1 (ELAVL1) [7, 8]. As a RNA-binding protein (RBP), ELAVL1 specifically binds to AU-rich elements (AREs) or U-rich elements (UREs), and participates in transcription, stabilization, and alternative splicing of pre-mRNAs [8]. However, the roles of *ELAVL1* in regulating alternative splicing events in gastric cancer remain elusive.

Small nucleolar RNAs (snoRNAs) are a highly conserved category of non-coding RNAs consisting of approximately 60 to 300 nucleotides and primarily localizing within nucleoli [9]. SnoRNAs are classified into C/D box, H/ACA box, or small Cajal body-associated ones [10, 11], and function as guide RNAs in the modification of small nuclear RNAs (snRNAs), ribosomal RNAs (rRNAs), and other cellular RNAs [10, 11]. In addition, snoRNAs may participate in pre-mRNA alternative splicing. For instance, *SNORD115* regulates alternative splicing of 5-hydroxytryptamine receptor 2C (*5-HT*_*2*_*CR*) pre-mRNA by interacting with its exon Vb [12]. *SNORA70E* facilitates RAS-related protein 1B (*RAP1B*)-mediated alternative splicing of poly (ADP-ribose) polymerase 1 binding protein (*PARPBP*) [13]. *SNORD2* participates in alternative splicing of its host gene eukaryotic translation initiation factor 4A2 (*EIF4A2*) [14]. Recent studies show the emerging roles of snoRNAs in the initiation and progression of tumors [13, 15], while their functions in gastric cancer remain to be determined.

Human *CD44* consists of 19 exons locating on chromosome 11p13, and serves as a biomarker for cancers [16]. The *CD44* standard isoforms (CD44s) are encoded by mature mRNA containing the first and last five exons, while those consisting of additional exons (exons 6–14) are collectively referred as *CD44* variant isoforms (CD44v) [5]. Specifically, exon 6 can be divided into two parts (exon 6A and 6B) that denote v1 and v2, respectively, while exons 7–14 are denoted as v3-v10 [5]. The inclusion of different exons and specific nomenclature of *CD44v* isoforms are related to tumor invasiveness [5, 17, 18]. For instance, *CD44v6* is increasingly expressed in colon cancer and pancreatic cancer, and is associated with worse prognosis of patients [17, 18]. In this study, we identify *SNORA37* as an *ELAVL1*-generated 129-nt H/ACA box snoRNA derived from host gene methyl-CpG binding domain protein 2 (*MBD2*), which is associated with progression and worse outcomes of gastric cancer. *SNORA37* directly binds to cap methyltransferase 1 (CMTR1) to facilitate its interaction with ELAVL1, resulting in nuclear retention and activity of ELAVL1 in regulating alternative splicing of *CD44*, which indicates the roles of *SNORA37*/CMTR1/ELAVL1 feedback loop in gastric cancer progression.

## Methods

### Cell culture

Human gastric cancer cell lines MKN-45 (JCRB0254), AGS (CRL-1739), HGC-27 (C6365), SNU-1 (CRL-5971), normal gastric mucosal GES-1 (C6268) cells, and embryonic kidney HEK293T cells (CRL-11268) were obtained from Japanese Collection of Research Bioresources Cell Bank (Osaka, Japan), American Type Culture Collection (Rockville, MD), or Beyotime Biotechnology (Beijing, China). Cell line authentication was performed by short tandem repeat (STR) profiling, and used within 6 months after resuscitation from frozen aliquots. Mycoplasma contamination was verified by using real-time quantitative RT-PCR (qRT-PCR) mycoplasma detection kit (Sigma, St. Louis, MO). The MKN-45, HGC-27, SNU-1, GES-1, and HEK293T cells were cultured in RPMI 1640 media (Gibco, Carlsbad, CA) supplemented with 10% fetal bovine serum (FBS, Gibco), while AGS was cultured in F12K (Gibco) media supplemented with 10% FBS (Gibco).

### RNA and protein isolation

Nuclear and cytoplasmic RNAs or proteins were isolated from cells following the protocol of Ambion^®^ PARIS™ Kit (Thermo Fisher Scientific, Waltham, MA). Briefly, the cells were initially washed with phosphate buffered solution (PBS), then resuspended in 100–500 μl of ice-cold Cell Fractionation Buffer and incubated on ice for 5–10 min. Subsequently, the samples were centrifuged for 1–5 min at 4 °C and 500 × g, after which the cytoplasmic fraction was carefully aspirated from nuclear pellet. The remaining fraction was homogenized in ice-cold Cell Fractionation Buffer to obtain the nuclear lysates. Finally, the sample was applied for RNA isolation or protein analysis. Total RNA was isolated following the manual of TRIzol reagent (Thermo Fisher Scientific) or QIAwave RNA Mini Kit (QIAGEN, Stockach, Germany), while total cellular or tissue proteins were extracted with RIPA lysis buffer (Thermo Fisher Scientific).

### RT-PCR and qRT-PCR

The cDNA was synthesized by using a reverse transcription (RT) kit with genomic DNA Eraser (Takara, Dalian, China). Quantitative analysis of mRNA and snoRNA was conducted using SYBR Green PCR kit (Takara), specific primers (Additional file 1: Table S1), and a StepOne real-time PCR system (Applied Biosystems, Carlsbad, CA). The levels of transcripts were normalized to those of glyceraldehyde-3-phosphate dehydrogenase (*GAPDH*) and determined by using the 2^−△△^Ct method [19, 20].

### RNA sequencing (RNA-seq)

Two micrograms of total RNA were utilized for stranded RNA library preparation, while transcriptome sequencing on Novaseq 6000 sequencer (Illumina) with PE150 model was performed at Seqhealth Tech Co. LTD (Wuhan, China). Then, the resulting data were filtered, aligned, and processed to calculate reads per kilobase (kb) of transcript per million mapped reads (RPKM). Alternative splicing events were detected by using rMATS (version 3.2.5) [21] with a false discovery rate (FDR) value less than 0.05. Percent spliced-in (PSI) was applied to quantify alternative splicing events by calculating the expression of individual exons in “exon inclusion” isoforms relative to total expression of all isoforms. Meanwhile, the difference of two PSI values of same gene, termed as delta-PSI (ΔPSI), across samples was used as an indicator of differential splicing. The ∆PSI threshold of 10% was considered to be statistically significant if *P-*value was less than 0.05 [22]. The sequencing results were submitted to Gene Expression Omnibus (GEO) under the accession number GSE285296 and GSE285402.

### Western blot assay

Western blot assay was carried out as previously documented [23–27], with antibodies against CMTR1 (ab70386), MBD2 (ab188474), Flag-tag (ab45766), myelin basic protein (MBP)-tag (ab40390), insulin-like growth factor 2 mRNA-binding protein 2 (IGF2BP2, ab124930), polypyrimidine tract-binding protein 1 (PTBP1, ab133734, Abcam, Cambridge, MA), ESF1 nucleolar pre-rRNA processing protein homolog (ESF1, 23496–1-AP), serine/arginine-rich splicing factor 1 (SRSF1, 12929–2-AP), nuclear valosin-containing protein-like (NVL, 16970–1-AP, Proteintech, Wuhan, China), hemagglutinin (HA)-tag (3724S), CD44 (3570S), heterogeneous nuclear ribonucleoprotein C (HNRNPC, 91327S), heterogeneous nuclear ribonucleoprotein U (HNRNPU, 34095S), splicing factor 3B subunit 1 (SF3B1, 14434S), U2 small nuclear RNA auxiliary factor 2 (U2AF2, 70471S), GAPDH (2118S, Cell Signaling Technology, Danvers, MA), ELAVL1 (sc-5261), histone H3 (sc-24516), or glutathione S-transferase (GST)-tag (sc-33614, Santa Cruz Biotechnology, Santa Cruz, CA).

### Gene over-expression and silencing

Human *SNORA37* cDNA (129 bp) was obtained by PCR amplification (Additional file 1: Table S2) and validated via Sanger sequencing. The expression vector of human *CMTR1* cDNA (2508 bp) was purchased from Genechem (Shanghai, China). Subsequently, *SNORA37* cDNA or *CMTR1* cDNA was subcloned into CV186 (Genechem), while *CMTR1* truncations were obtained via PCR amplification and primer sets (Additional file 1: Table S2), and inserted into pGEX-6P-1 or pCMV-HA (Addgene, Cambridge, MA), respectively. The *ELAVL1* vectors were established as previously described [28], while MBP tagged-*ELAVL1* truncations were obtained via PCR amplification and primer sets (Additional file 1: Table S2), and inserted into pMAL-c4X (Addgene). Oligonucleotides specific for short hairpin RNAs (shRNAs) targeting *SNORA37*, *CMTR1*, or *ELAVL1* (Additional file 1: Table S3) were inserted into the GV298 (Genechem). The constructs were transfected using NEOFECT^®^ DNA transfection reagent (NEOFECT, Beijing, China), and stable cancer cell lines were established by selection with puromycin (Invitrogen, Carlsbad, CA).

### Rescue of target gene expression

To rescue *SNORA37* silencing-altered gene expression, *CMTR1* or *ELAVL1* construct was transfected into stable cell lines with NEOFECT^®^ DNA transfection reagent (NEOFECT). To restore gene expression induced by *SNORA37* over-expression, shRNAs against *CMTR1* or *ELAVL1* (Additional file 1: Table S3) were transfected into stable cancer cells with NEOFECT^®^ DNA transfection reagent (NEOFECT).

### RNA fluorescence in situ hybridization (FISH)

By utilizing a double-stranded DNA template containing a T7 promoter consensus motif (Additional file 1: Table S1) and biotin-16-UTP (Roche, Basel, Switzerland), the probes for *SNORNA37*, *GAPDH*, or *U1* were synthesized, while antisense probe for *SNORA37* was prepared with In vitro Transcription T7 Kit (Takara). Subsequent RNA purification was conducted by using RNeasy Min Elute Cleanup Kit (QIAGEN). Hybridization was performed using FISH kit (RiboBio, Guangzhou, China) following the manufacturer’s instructions, with nuclei counterstained using 4',6-diamidino-2-phenylindole (DAPI). The images were observed and photographed under a Nikon A1Si Laser Scanning Confocal Microscope (Nikon, Japan) [20, 25].

### Biotin-labeled RNA pull-down and mass spectrometry

The biotin-labeled *SNORA37* sense and antisense probes were synthesized by aforementioned in vitro transcription method, and incubated overnight with streptavidin magnetic beads, cell lysates, and RNAase inhibitors at 4℃. The bound proteins were subsequently retrieved for silver staining detection through Pierce Silver Stain Kit (Thermo Fisher Scientific) [20, 25], while other portion was subjected to mass spectrometry analysis at Wuhan SpecAlly Tech Co. LTD (Wuhan, China).

### Cross-linking RNA immunoprecipitation (RIP)

At 254 nm (200 J/cm^2^), ultraviolet light was used to cross-link cells [20, 25]. RIP assay was conducted according to the protocol provided by Magna RIP™ RNA-Binding Protein Immunoprecipitation Kit (Millipore, Bedford, MA). Briefly, cells were lysed in RIP lysis buffer, and incubated with magnetic beads and antibodies specific to CMTR1 (ab70386), HA-tag (3724S), GST-tag (sc-33614), ELAVL1 (sc-5261), HNRNPC (91327S), HNRNPU (34095S), or PTBP1 (ab133734) at 4 °C overnight [20, 25]. Following washing, RNA was purified using RNeasy MinElute Cleanup Kit (QIAGEN), and co-precipitated RNA was detected by RT-PCR or real-time qRT-PCR with specific primers (Additional file 1: Table S1).

### In vitro binding assay

The GST-tagged *CMTR1* (pGEX-6P-1) or MBP-tagged *ELAVL1* (pMAL-c4X) truncation constructs were transformed into *E. coli* BL21 strain (Thermo Fisher Scientific), while proteins were purified by Pierce GST Spin Purification Kit or Pro-Detect™ Rapid MBP Assay Kit (Thermo Fisher Scientific). The *SNORA37* was transcribed by using In vitro Transcription T7 Kit (Takara) and purified with RNeasy Min Elute Cleanup Kit (QIAGEN) [20, 25], followed by co-incubation with GST-tagged CMTR1 or MBP-tagged ELAVL1 protein. The anti-GST beads (Sigma) were used to pull down protein-RNA complex. Protein was then detected through sodium dodecyl sulfate (SDS)-polyacrylamide gel electrophoresis (PAGE) and western blot, while RNA was measured through RT-PCR using specific primers (Additional file 1: Table S1).

### Fluorescence immunocytochemical staining

Cancer cells were grown on confocal dish, and incubated with complete medium until fully adherent. Then, cells were with 4% paraformaldehyde and treated with antibodies specific for CMTR1 (ab70386, Abcam; 1:200 dilution) or ELAVL1 (sc-5261, 1:100 dilution) at 4 °C overnight. Then, coverslips were treated with Cy3-conjugated goat anti-rabbit IgG (1:1000 dilution) or 488-conjugated goat anti-rabbit IgG, and stained by DAPI (300 nmol/L). The images were photographed under a Nikon A1Si Laser Scanning Confocal Microscope (Nikon) [19, 20, 25–27].

### Co-immunoprecipitation (Co-IP)

Co-IP experiment was conducted as previously reported [19, 20, 25–29], utilizing specific antibodies against CMTR1 (ab70386), ELAVL1 (sc-5261), Flag-tag (ab45766), MBP (ab40390), HA-tag (3724S, Cell Signaling Technology), or GST (sc-33614, Santa Cruz Biotechnology) for immunoprecipitation. Subsequently, proteins bound to Protein A/G Magnetic Beads (MedChemExpress, shanghai, China) were recovered and detected by western blot.

### Bimolecular fluorescence complementation (BiFC) assay

Human *CMTR1* cDNA (2508 bp) and *ELAVL1* cDNA (1818 bp) were respectively inserted into pBiFC-VC155 (22011, Addgene) or pBiFC-VN173 (22010, Addgene; Additional file 1: Table S2). After co-transfection of these constructs by using NEOFECT^®^ DNA transfection reagent (NEOFECT) for 24 h, cells were fixed with 4% paraformaldehyde, stained with DAPI for 10 min, and observed under a Nikon A1Si Laser Scanning Confocal Microscope (Nikon). The excitation and emission wavelengths of confocal microscope were 488 nm and 500 nm, respectively [19, 20, 25–27].

### In vitro cellular viability, growth, and invasion assays

In vitro viability, growth, and invasive capabilities of cancer cells were detected by 2-(4,5-dimethyltriazol-2-yl)-2,5-diphenyl tetrazolium bromide (MTT, Sigma) colorimetric [30], soft agar (Noble agar, Sigma) [19, 20, 25–27], and matrigel (BioCoat™ Matrigel®, Corning, NY, USA) invasion assays, respectively [19, 20, 25–27].

### Xenografts in nude mice

All animal experiments were conducted in accordance with the guidelines established by Experimental Animal Ethics, Huazhong University of Science and Technology and National Institutes of Health’s Guidelines for the Care and Use of Experimental Animals. Subcutaneous xenograft and tail vein metastasis experiments were performed on 4-week-old BALB/c nude mice (National Rodent Seed Center, Shanghai, China), with injection of 1 × 10^6^ cancer cells for subcutaneous xenograft studies, or 1 × 10^7^ cancer cells for tail vein metastasis experiment, respectively [19, 20, 25–27]. The condition of nude mice was monitored daily, and their tumor volume, body weight, time of death, and survival were recorded. Subcutaneous xenograft mice were euthanized at 4 weeks after initial injection of cancer cells. Experimental mice with tail vein metastasis were euthanized 7 weeks after initial injection of cancer cells, and their lung or liver tissues were dissected. The in vivo Xtreme II small animal imaging system (Bruker, Billerica, MA, USA) was used for imaging [19, 20, 25–27].

### Clinical tissues

Human tissue study was approved by the Institutional Review Board of Union Hospital, Tongji Medical College, and conducted in accordance with the guidelines of Declaration of Helsinki. Gastric cancer and adjacent normal epithelial tissues were collected during surgery at Union Hospital, Tongji Medical College, and validated by pathological diagnosis. Written informed consent was obtained from all patients. Fresh tumor tissues were preserved in RNAsafer Stabilizer Reagent (Omega, Guangzhou, China), frozen in liquid nitrogen, and stored at -80 °C.

### Immunohistochemistry staining

Immunohistochemical staining and quantitative evaluation were performed as previously described [19, 20, 25–28], with antibodies specific for CD31 (ab28364, Abcam; 1:100 dilution) or Ki-67 (sc-23900, Santa Cruz Biotechnology; 1:100 dilution). The degree of positivity was measured according to the percentage of positive cancer cells.

### Statistical analysis

All data were shown as mean ± standard deviation (SD). Cutoff values were determined by medium gene expression levels. Student′s *t*-test, Mann–Whitney U test, and analysis of variance (ANOVA) were applied to compare differences in cancer cells or tissues [31]. Statistical significance of overlap analysis was determined by Fisher′s exact test. Log-rank test was used to assess survival differences. All statistical tests were two-sided [31].

## Results

### *SNORA37* is associated with unfavorable prognosis of gastric cancer

**Fig. 1:**
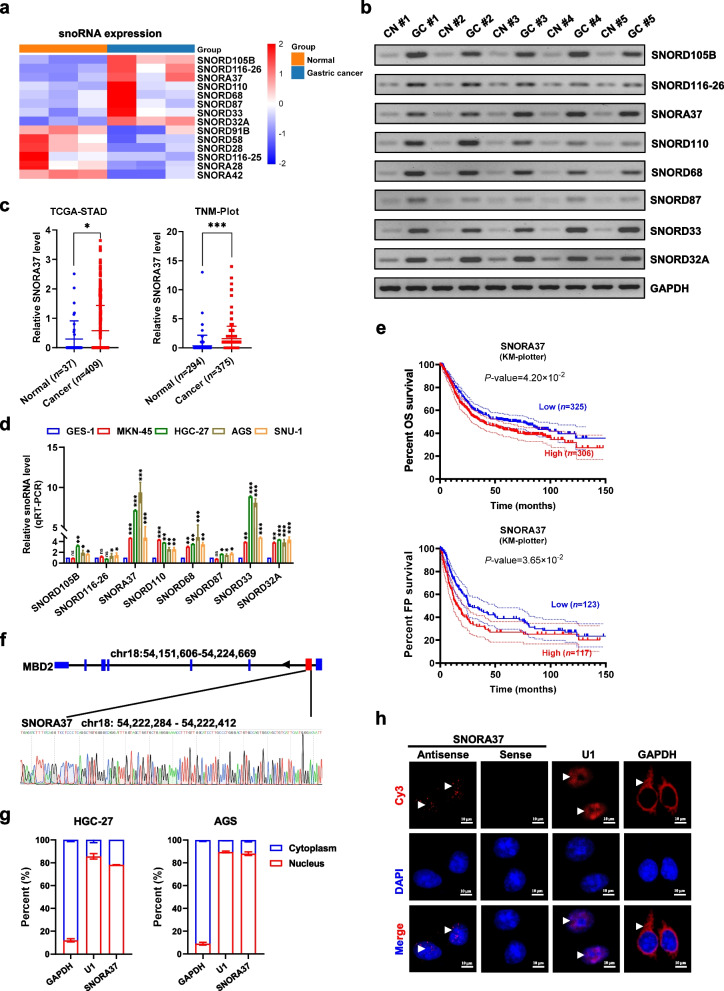
*SNORA37* is associated with unfavorable prognosis of gastric cancer. **a** Heatmap illustrating the differential expression (fold change ≥ 2.0, *P-*value < 0.05) of snoRNAs between three pairs of pathological validated gastric cancer and corresponding normal epithelial tissues. **b** Validating RT-PCR assay showing the expression levels of eight snoRNAs in gastric cancer (GC) and corresponding normal (CN) epithelial tissues. *GAPDH* served as a control. **c** The expression levels of *SNORA37* in normal gastric epithelia and gastric cancer tissues derived from TCGA (https://cancergenome.nih.gov) and TNM-plot (https://tnmplot.com/) databases. **d** Real-time qRT-PCR assay showing the relative expression of snoRNAs (normalized to *GAPDH*, *n* = 4) in GES-1, MKN-45, HGC-27, AGS, and SNU-1 cells. **e** Kaplan–Meier curves indicating overall survival (OS) and first progression (FP) survival of gastric cancer cases with low or high levels of *SNORA37* (cutoff values = 4.95 and 5.08, respectively). **f** Schematic representation showing genomic location of *SNORA37* derived from its host gene *MBD2*, and validation by Sanger sequencing. **g** Real-time qRT-PCR indicating the distribution of *SNORA37*, *GAPDH*, and *U1* in cytoplasmic and nuclear fractions of HGC-27 and AGS cells (*n* = 4). **h** RNA-FISH assay visualizing the cytoplasmic and nuclear localization of *SNORA37* (red, arrowheads) in HGC-27 cells, with nuclei staining with DAPI (blue). *SNORA37* sense probe was used as a negative control. *GAPDH* and *U1* were used as positive controls. Non-parametric Mann–Whitney U test compared the difference in **c**. One-way analysis of variance (ANOVA) compared the difference in **d**. Log-rank test was used for survival comparison in **e**. *P < 0.05, ** *P* < 0.01, *** *P* < 0.001 vs. normal or GES-1. ns, non-significant. Data are shown as mean ± s.e.m. (error bars) and representative of three independent experiments in **b**, **d**, **g** and **h**

To identify snoRNAs associated with gastric cancer progression, high-throughput RNA-seq was performed by using three pairs of cancerous and corresponding normal epithelial specimens. The results revealed 14 differentially expressed snoRNAs (fold change ≥ 2.0, *P-*value < 0.05) in gastric cancer tissues, including 8 up-regulated and 6 down-regulated snoRNAs (Fig. 1a and Additional file 1: Table S4). Elevated expression of eight snoRNAs was validated by RT-PCR in gastric cancer tissues (Fig. 1b). Furthermore, analysis of public datasets from The Cancer Genome Atlas (TCGA, https://cancergenome.nih.gov) or Tumor-Normal-Metastatic-plot (TNM-plot, https://tnmplot.com/) database revealed that *SNORD105B*, *SNORD116-26*, *SNORA37*, *SNORD110*, *SNORD68*, *SNORD33*, and *SNORD32A* levels were also elevated in gastric cancer tissues than those in normal gastric epithelia (Fig. 1c and Additional file 1: Fig. S1a). In cultured gastric cancer cell lines (MKN-45, HGC-27, AGS, SNU-1), the expression of *SNORA37* and *SNORD33* was consistently enhanced than that of GES-1 cells (Fig. 1d). Survival analysis with Kaplan–Meier Plotter (KM-Plotter) program (https://kmplot.com) indicated that only *SNORA37* was significantly associated with poor overall survival (OS) and first progression (FP) survival of gastric cancer patients (Fig. 1e), which was chosen for further studies. Nuclear-cytoplasmic fractionation and RNA-FISH assays indicated that *SNORA37*, a H/ACA box snoRNA derived from intron 1 of host gene *MBD2* (Fig. 1f), was predominantly localized within the nucleus of HGC-27, AGS, MKN-45, and SUN-1 cells (Fig. 1g, h and Additional file 1: Fig. S1b). To explore mechanisms underlying *SNORA37* biogenesis, 10 RBPs binding to 1 kb upstream or downstream of its genomic region (chr18: 54222284–54222412) were identified by analyzing public crosslinking-immunoprecipitation and high-throughput sequencing (CLIP-seq) datasets derived from POSTAR 3.0 (http://111.198.139.65/) and ENCORI (https://rnasysu.com/encori/) databases (Additional file 1: Fig. S1c). Further analysis of datasets derived from KM-Plotter database revealed that 6 RBPs were significantly associated with survival of gastric cancer patients (Additional file 1: Fig. S1c). Among them, ELAVL1, an essential splicing factor [8], was the only RBP associated with poor outcomes of gastric cancer (Additional file 1: Fig. S1c). Enrichment of ELAVL1 was noted within upstream region of *SNORA37* (Additional file 1: Fig. S1c). Cross-linking RIP assay revealed endogenous binding of ELAVL1 to intron 1 of *MBD2* pre-mRNA in gastric cancer cell lines HGC-27 and AGS, which was attenuated by knockdown of *ELAVL1* (Additional file 1: Fig. S1c). Moreover, silencing of *ELAVL1* decreased the levels of *SNORA37*, without alteration of *MBD2* expression, in gastric cancer cells (Additional file 1: Fig. S1c). These results indicated that *SNORA37* was associated with unfavorable prognosis of gastric cancer.

### *SNORA37* promotes tumorigenesis and aggressiveness of gastric cancer

**Fig. 2:**
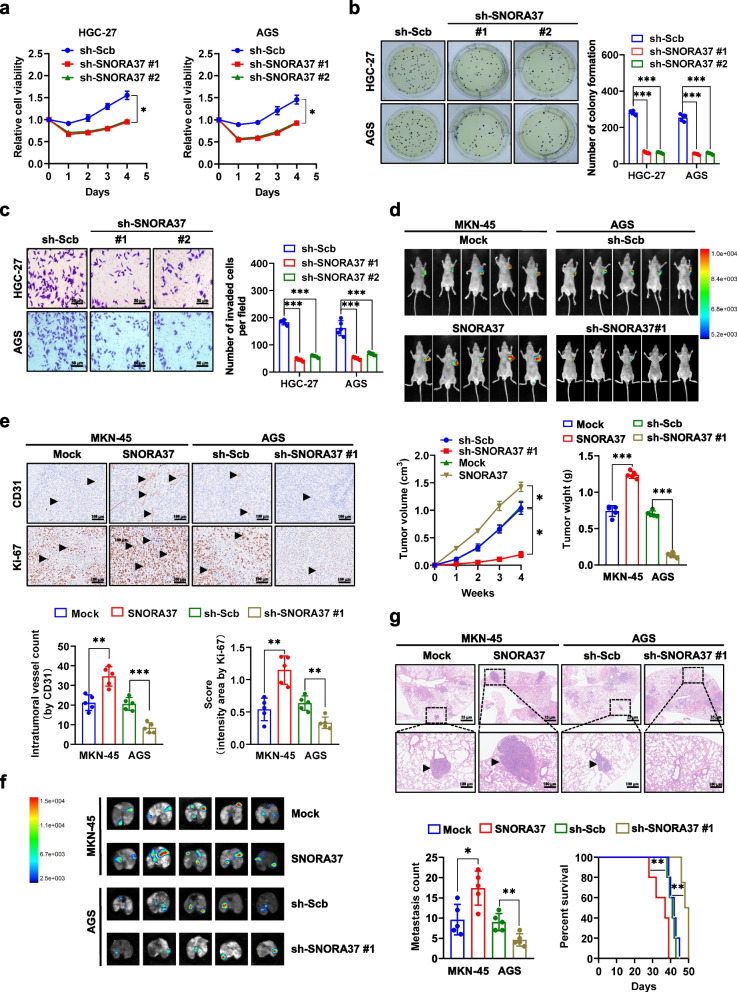
*SNORA37* promotes tumorigenesis and aggressiveness of gastric cancer. **a** MTT colorimetric assay showing the relative viability of HGC-27 and AGS cells stably transfected with scramble shRNA (sh-Scb) or sh-SNORA37 #1 (*n* = 5). **b** and **c** Representative images (left panel) and quantification (right panel) of soft agar (**b**) and matrigel invasion (**c**) assays indicating the growth and invasion of HGC-27 and AGS cells stably transfected with sh-Scb, sh-SNORA37 #1, or sh-SNORA37 #2 (*n* = 5). **d** In vivo imaging, growth curve, and weight at the endpoints of xenograft tumors formed by subcutaneous injection of MKN-45 cells stably transfected with empty vector (mock) or *SNORA37*, or AGS cells stably transfected with sh-Scb or sh-SNORA37 #1 into dorsal flanks of nude mice (*n* = 5 for each group). **e** Representative images (upper panel) and quantification (lower panel) of immunohistochemical staining showing the intertumoral expression of CD31 and Ki-67 (brown, arrowheads) within subcutaneous xenograft tumors of nude mice formed by MKN-45 or AGS cells stably transfected with mock, *SNORA37*, sh-Scb or sh-SNORA37 #1 (*n* = 5 for each group). **f** and **g** Representative images (**f**), hematoxylin & eosin (HE) staining (g, upper panel), quantification (g, bottom left panel) of lung metastatic colonization (arrowheads) and Kaplan–Meier curves (g, bottom right panel) of nude mice treated with tail vein injection of MKN-45 or AGS cells stably transfected with mock, *SNORA37*, sh-Scb or sh-SNORA37 #1 (*n* = 5 for each group). One-way analysis of variance (ANOVA) was used to compare the difference in **a**-**d**. Student's *t*-test compared the difference in **d**, **e** and **g**. Log-rank test was used for survival comparison in **g**. *P < 0.05, ** *P* < 0.01, *** *P* < 0.001. Data are shown as mean ± s.e.m. (error bars) and representative of three independent experiments in **a-c**

To explore the roles of *SNORA37* in gastric cancer progression, MKN-45 and SNU-1 cells (with relatively moderate *SNORA37* levels) were chosen as models for stable over-expression studies, while HGC-27 and AGS cell lines (with relatively high *SNORA37* expression) were applied for stable knockdown experiments (Additional file 1: Fig. S2a). In MTT colorimetric assay, stable over-expression or silencing of *SNORA37* enhanced or reduced the viability of gastric cancer cells (Additional file 1: Fig. S2b and Fig. 2a). Soft agar and matrigel invasion assays revealed the increase or decrease of anchorage-independent growth and invasiveness of gastric cancer cells with stable over-expression or silencing of *SNORA37* (Additional file 1: Fig. S2c, d and Fig. 2b, c). In addition, there were significant increases or decreases in volume, weight, microvascular density, and proliferation index of xenograft tumors in nude mice generated by subcutaneous injection of MKN-45 or AGS cells stably transfected by *SNORA37* or shRNA targeting *SNORA37* (sh-SNORA37; Fig. 2d, e and Additional file 1: Fig. S2e). However, following over-expression or knockdown of *SNORA37*, no significant changes were observed in the transcriptional or protein levels of host gene *MBD2* within cultured cell lines or xenograft tumors (Additional file 1: Fig. S2f, g). Notably, intravenous injection of MKN-45 cells with stable over-expression of *SNORA37* led to more lung or liver metastasis and poorer survival in nude mice (Fig. 2f, g and Additional file 1: Fig. S2h). Conversely, intravenous injection of AGS cells with stable knockdown of *SNORA37* resulted in fewer lung or liver metastasis colonies and higher survival potential (Fig. 2f, g and Additional file 1: Fig. S2h). Collectively, these findings indicated that *SNORA37* promoted tumorigenesis and aggressiveness of gastric cancer.

### *SNORA37* facilitates alternative splicing of oncogenes *CD44* and *PRMT2*

**Fig. 3:**
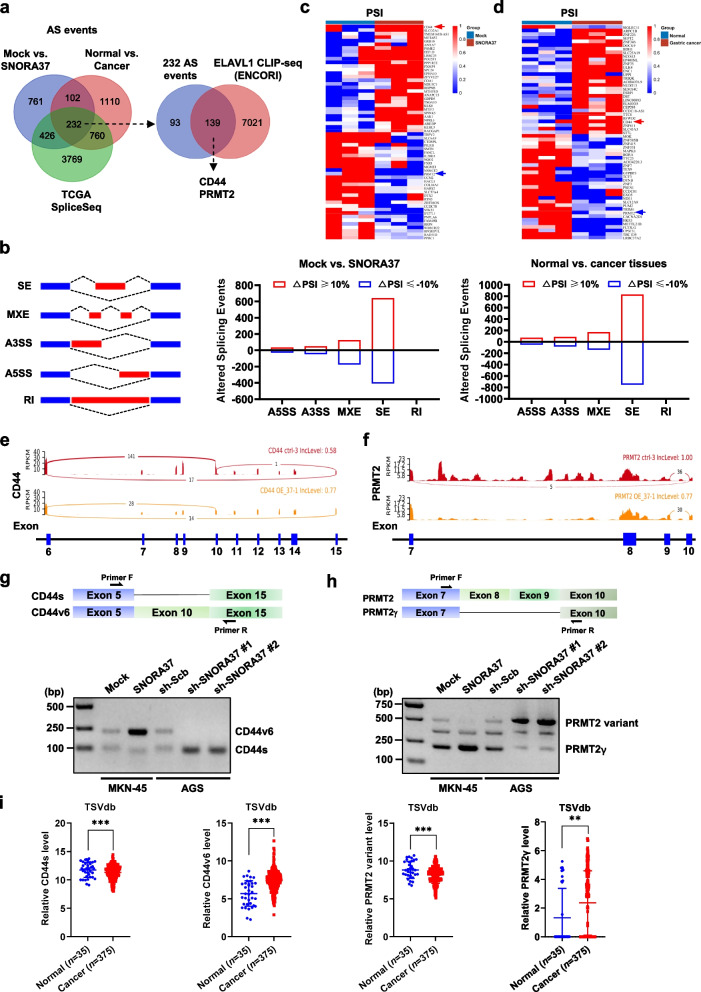
*SNORA37* facilitates alternative splicing of oncogenes *CD44* and *PRMT2*. **a**, Venn diagram revealing the overlapping analysis of changes in alternative splicing (AS) genes (|ΔPSI|≥ 10%, *P* < 0.05) in MKN-45 cells stably transfected with empty vector (mock) or *SNORA37* (*n* = 3), those between gastric cancer and adjacent normal epithelial tissues (*n* = 3), AS events identified in TCGA SpliceSeq (https://bioinformatics.mdanderson.org/TCGASpliceSeq) database, and ELAVL1 downstream targets in CLIP-seq dataset derived from ENCORI (https://rnasysu.com/encori/) database, with *CD44* and *PRMT2* exhibiting significant AS events.** b**, Schematic diagram (left panel) showing primary five types of AS patterns, including skipping exon (SE), mutually exclusive exons (MXE), alternative 3' splice sites (A3SS), alternative 5' splice sites (A5SS), and retained intron (RI). The number of altered AS events in MKN-45 cells stably transfected with mock or *SNORA37* (middle panel), and that between gastric cancer and adjacent normal epithelial tissues (right panel). **c** and **d**, Heatmap illustrating top 60 changes in AS events (|ΔPSI|≥ 50%, *P* < 0.05) in MKN-45 cells stably transfected with mock or *SNORA37* (c, *n* = 3), and those between gastric cancer and adjacent normal epithelial tissues (d, *n* = 3). **e** and **f**, Sashimi plot illustrating the splicing in exon 10 of *CD44* (e) and exons 7–10 of *PRMT2* (f) in MKN-45 cells stably transfected with mock or *SNORA37*. **g** and **h**, Schematic diagram (upper panel) and RT-PCR assay (lower panel) indicating the differential expression levels of *CD44v6*, *CD44s*, *PRMT2* variant, or *PRMT2γ* in MKN-45 and AGS cells stably transfected with mock, *SNORA37*, sh-Scb, sh-SNORA37 #1, or sh-SNORA37 #2. **i**, Relative splicing events of *CD44s*, *CD44v6*, *PRMT2* variant, or *PRMT2γ* in normal gastric epithelia (*n* = 35) and tumor tissues (*n* = 375) of gastric cancer cases derived from TSVdb database (http://www.tsvdb.com). Fisher's exact test for over-lapping analysis in **a**. Non-parametric Mann–Whitney U test compared the difference in **i**. ** *P* < 0.01, *** *P* < 0.001. Data are shown as representative of three independent experiments in **g** and **h**

To investigate downstream targets of *SNORA37*, RNA-seq was performed to reveal 1521 alternative splicing events [|ΔPSI|≥ 10%, *P* < 0.05] in MKN-45 cells upon *SNORA37* over-expression (Fig. 3a). Meanwhile, 2204 alternative splicing events were also discovered in gastric cancer tissues compared to those in adjacent normal epithelial tissues (Fig. 3a). Through comprehensive analysis of these alternative splicing events with those identified in TCGA SpliceSeq database (https:// bioinformatics.mdanderson.org/TCGASpliceSeq), 232 common genes were noted (Fig. 3a), while 139 of them were ELAVL1 downstream targets in CLIP-seq dataset derived from ENCORI database, including *CD44* and protein arginine methyltransferase 2 (*PRMT2*, Fig. 3a). Among five main patterns (SE, MXE, A3SS, A5SS, and RI) [4, 5], SE-type alternative splicing events were most prevalent in both *SNORA37*-overexpressing cancer cells and gastric tissues (Fig. 3b). Based on over-lapping analysis of top 60 changes in alternative splicing events (|ΔPSI|≥ 50%, *P* < 0.05) in MKN-45 cells over-expressing *SNORA37* (Fig. 3c, Additional file 1: Table S5) and gastric cancer tissues (Fig. 3d, Additional file 1: Table S6), only *CD44* and *PRMT2* were found to exhibit consistent alternative splicing events and selected for further analysis. Both *CD44* and *PRMT2* were up-regulated in gastric cancer tissues (Additional file 1: Fig. S3a), and associated with unfavorable survival of patients (Additional file 1: Fig. S3b). Specially, RNA-seq, validating RT-PCR, and Sanger sequencing assays indicated that splicing of *CD44v6* and *PRMT2γ* variants were enhanced or reduced in MKN-45 or AGS cells following *SNORA37* over-expression or silencing, along with decrease or increase in *CD44s* and *PRMT2* variants levels (Fig. 3e-h and Additional file 1: Fig. S3c, d). Notably, search of TSVdb database (http://www.tsvdb.com/) revealed the up-regulation of *CD44v6* or *PRMT2γ* and down-regulation of *CD44s* or *PRMT2* variant in gastric cancer tissues (Fig. 3i). These findings indicated that *SNORA37* facilitated alternative splicing of oncogenes *CD44* and *PRMT2* in gastric cancer.

### *SNORA37* directly interacts with nuclear CMTR1 essential for *CD44* alternative splicing and aggressiveness of gastric cancer

**Fig. 4:**
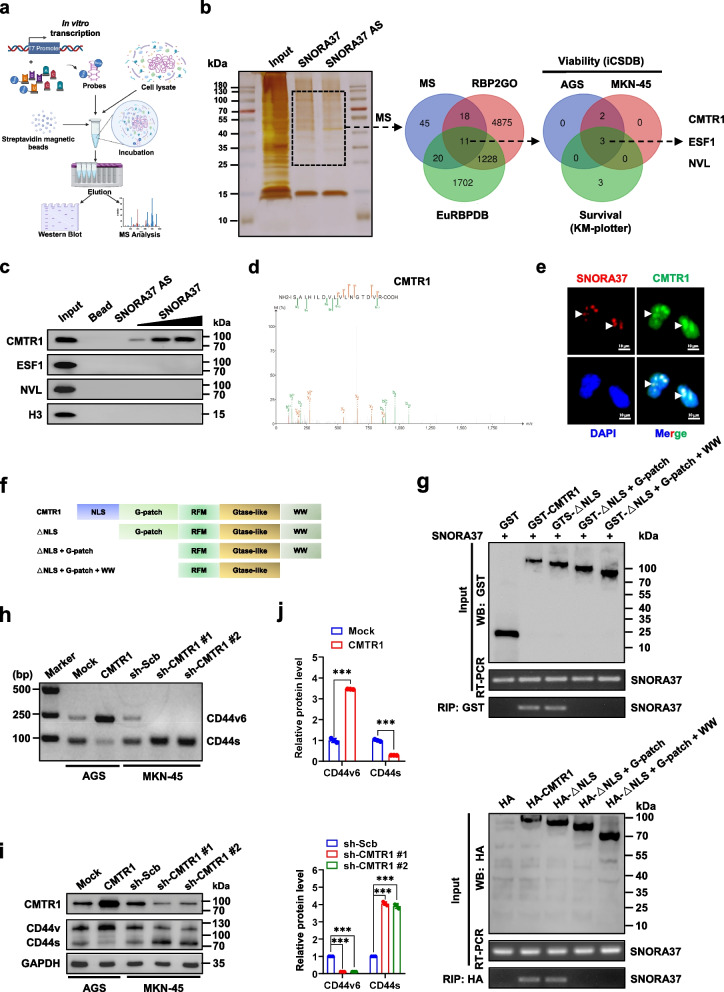
*SNORA37* directly interacts with nuclear CMTR1 essential for *CD44* alternative splicing and aggressiveness of gastric cancer. **a** Schematic illustration showing the identification of *SNORA37*-binding proteins. **b** SDS-PAGE, silver staining (left panel), mass spectrometry (MS) assay, and Venn diagram (middle panel) indicating the differential proteins pulled down by biotin-labeled sense or antisense (AS) *SNORA37* from AGS cells, and overlapping analysis with established RBPs from EuRBPDB (http://eurbpdb.gzsys.org.cn) and RBP2GO (https:// rbp2go.dkfz.de) databases. Further over-lapping (right panel) identifying the RBPs essential for both cellular viability and patients’ survival of gastric cancer by using datasets derived from iCSDB (https://www.kobic.re.kr/icsdb/) and KM-Plotter (http://kmplot.com) databases. **c** Biotin-labeled RNA pull-down and western blot assays showing the proteins pulled down by biotin-labeled *SNORA37* from HGC-27 cell lysates, using *SNORA37* antisense transcript or beads as controls. **d** Mass spectrometry assay revealing CMTR1 peptide fragment pulled down by *SNORA37*. **e** Dual RNA-FISH and immunofluorescence staining assays indicating the co-localization of *SNORA37* (red, arrowheads) and CMTR1 (green, arrowheads) in HGC-27 cells, with nuclei staining with DAPI (blue). **f** Schematic diagram revealing the domains of *CMTR1* truncations. **g** In vitro binding (upper panel) and RIP (lower panel) assays showing the enrichment of *SNORA37* detected by RT-PCR after incubation with GST-tagged recombinant CMTR1 proteins, or lysates of AGS cells transfected with full-length or truncations of HA-tagged *CMTR1* constructs validated by western blot. **h** RT-PCR assay indicating the differential expression levels of *CD44v6* and *CD44s* in gastric cancer AGS and MKN-45 cells stably transfected with empty vetor (mock), *CMTR1*, scramble shRNA (sh-Scb), sh-CMTR1 #1, or sh-CMTR1 #2. **i** and** j** Western blot assay (i) and quantification (j) indicating the differential expression levels of *CD44v6* and *CD44s* in AGS and MKN-45 cells stably transfected with mock, *CMTR1*, sh-Scb, sh-CMTR1 #1, or sh-CMTR1 #2 (*n* = 3). Fisher's exact test for over-lapping analysis in **b**. Student's *t*-test or one‐way ANOVA analyzed the difference in **j**. ***P < 0.001. Data are shown as mean ± SEM (error bars) and representative of three independent experiments in **c**, **e** and **g**-**j**

To discover protein partner of *SNORA37*, biotin-labeled RNA pull-down assay was conducted using AGS cell lysates, followed by mass spectrometry (MS) analysis (Fig. 4a). The results revealed 94 differentially expressed proteins between *SNORA37* and its antisense transcript pull-down groups (Additional file 1: Table S7), out of which 11 candidates were RBPs consistently defined in EuRBPDB (http://eurbpdb.gzsys.org.cn) and RBP2GO (https://rbp2go.dkfz.de) databases (Fig. 4b). Through further comprehensive analysis of gastric cancer datasets derived from Integrated Database of CRISPR Screens (iCSDB, https://www.kobic.re.kr/icsdb/) [32] and KM-Plotter database, CMTR1, ESF1, and NVL were chosen as potential *SNORA37*-binding proteins essential for both cellular viability and patients’ survival of gastric cancer (Fig. 4b). To reveal consistent *SNORA37*-binding protein, another gastric cancer cell line HGC-27 was applied. Validating RNA pull-down and western blot assays indicated the presence of CMTR1, but not of ESF1 or NVL, in HGC-27 cell lysates pulled down by *SNORA37* (Fig. 4c, d). Consistently, co-localization of *SNORA37* and CMTR1 was observed in the nucleus of HGC-27 cells (Fig. 4e). Cross-linking RIP assay also revealed the interaction of CMTR1 with *SNORA37* in HGC-27, AGS, MKN-45, and SNU-1 cells (Additional file 1: Fig. S4a). In vitro binding, RIP, and western blot assays indicated that G-patch domain, but not nuclear localization signal (NLS), Rossman-fold methyltransferase domain (RFM), Gtase-like, or WW domain, of GST- or HA-tagged CMTR1 protein was crucial for its interaction with *SNORA37* (Fig. 4f, g). Of note, ectopic expression or silencing of *CMTR1* facilitated or reduced the splicing and protein expression of *CD44v6* in AGS or MKN-45 cells (Additional file 1: Fig. S4b and Fig. 4h-j). These findings suggested that *SNORA37* directly interacted with nuclear CMTR1 essential for *CD44* alternative splicing and aggressiveness of gastric cancer.

### CMTR1 interacts with splicing factor ELAVL1 in gastric cancer cells

**Fig. 5:**
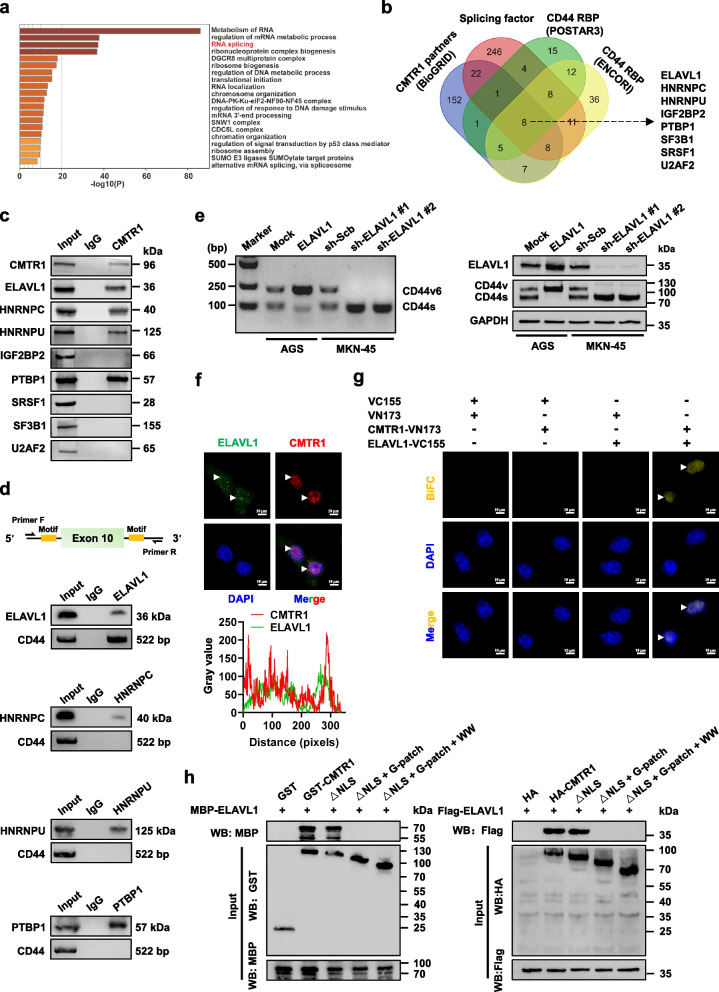
CMTR1 interacts with splicing factor ELAVL1 in gastric cancer cells. **a** GO pathway analysis via Metascape program (https://metascape.org) of 204 CMTR1-interacting proteins derived from BioGRID database (https://thebiogrid.org). **b** Venn diagram showing the identification of CMTR1-binding partners via over-lapping analysis of CMTR1-interactng proteins derived from BioGRID database, established splicing factors, and RBPs binding with *CD44* pre-mRNA in POSTAR3 (http://postar.ncrnalab.org) or ENCORI (https://rnasysu.com/encori/) database. **c** Co-IP and western blot assays indicating the interaction of CMTR1 with splicing factors in AGS cells. **d** Schematic depiction (upper panel) and cross-linking RIP assay (lower panel) showing the interaction of ELAVL1, HNRNPC, HNRNPU, or PTBP1 with *CD44* pre-mRNA containing alternative splicing sites around exon 10 in AGS cells. **e** RT-PCR assay (left panel) and western blot (right panel) assays indicating the differential expression levels of *CD44v6* and *CD44s* in gastric cancer AGS and MKN-45 cells stably transfected with empty vector (mock), *ELAVL1*, scramble shRNA (sh-Scb), sh-ELAVL1 #1, or sh-ELAVL1 #2. **f** Representative images (upper panel) and quantification (lower panel) of immunofluorescence assay showing the co-localization (arrowheads) of ELAVL1 with CMTR1 in HGC-27 cells. **g** BiFC assay indicating interaction between CMTR1 and ELAVL1 (arrowheads) within HGC-27 cells co-transfected with pBiFC-VN173-CMTR1 and pBiFC-VC155- ELAVL1, with nuclei staining by DAPI. **h** In vitro binding (left panel), co-IP (right panel) and western blot assays showing the interaction between GST-tagged CMTR1 and MBP-tagged ELAVL1 proteins, and that in AGS cells transfected with full-length or truncations of HA-tagged *CMTR1* and Flag-tagged *ELAVL1* constructs. Fisher's exact test for over-lapping analysis in **b**. Data are representative of three independent experiments in **c**-**h**

To investigate the mechanisms underlying CMTR1-mediated alternative splicing, its interacting proteins were obtained from BioGRID database (https://thebiogrid.org) and subjected to Gene Ontology (GO) pathway analysis using Metascape program (https://metascape.org, Fig. 5a). The results unveiled a substantial number of potential partners involved in RNA splicing (Fig. 5a). Further over-lapping analysis with splicing factors [4] and *CD44* pre-mRNA-binding RBPs derived from POSTAR3 (http://postar.ncrnalab.org) and ENCORI databases indicated 8 potential proteins involved in this process (Fig. 5b). Co-IP and western blot assays indicated the interaction of CMTR1 with ELAVL1, HNRNPC, HNRNPU, or PTBP1, but not with other proteins, in AGS cell (Fig. 5c). Intriguingly, only ELAVL1 was able to bind with intronic regions surrounding exon 10 of *CD44* (Fig. 5d and Additional file 1: Fig. S5a, b). Ectopic expression or knockdown of *ELAVL1* led to increase or decrease in the splicing and protein expression of *CD44v6* variant (Fig. 5e). Of importance, endogenous co-localization of CMTR1 and ELAVL1 was observed in the nucleus of HGC-27 cells (Fig. 5f), which was facilitated by ectopic expression of *CMTR1* (Additional file 1: Fig. S5c). In addition, BiFC assay confirmed the interaction between CMTR1 and ELAVL1 in HGC-27 cells (Fig. 5g). In vitro binding and western blot assays indicated that G-patch domain, but not NLS, RFM, Gtase-like, or WW domain, of GST-tagged recombinant CMTR1 protein was required for its interaction with MBP-tagged ELAVL1 (Fig. 5h). Similarly, Hinge domain, but not RNA recognition motif 1 (RRM1), RRM2, or RRM3 domain, of MBP-tagged recombinant ELAVL1 protein was required for its interaction with GST-tagged CMTR1 (Additional file 1: Fig. S5d), which was further confirmed by co-IP and western blot assays of AGS cells transfected with HA-tagged *CMTR1* or Flag-tagged *ELAVL1* truncations (Fig. 5h, Additional file 1: Fig. S5d). Of note, over-expression or silencing of *CMTR1* led to elevation or reduction in nuclear retention of ELAVL1 in AGS cells (Additional file 1: Fig. S5e). These data indicated that CMTR1 interacted with splicing factor ELAVL1 in gastric cancer cells.

### *SNORA37* facilitates CMTR1-ELAVL1 interplay in alternative splicing of *CD44*

**Fig. 6:**
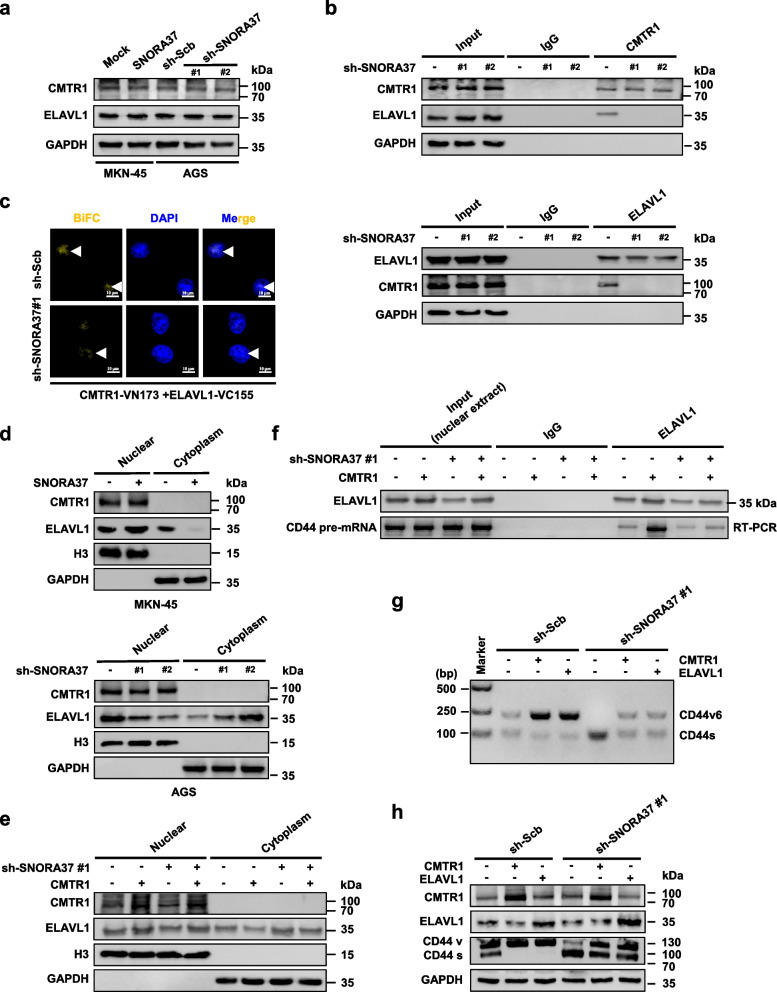
*SNORA37* facilitates CMTR1-ELAVL1 interplay in alternative splicing of *CD44*. **a** Western blot assay showing the expression of CMTR1 and ELAVL1 in MKN-45 and AGS cells stably transfected with empty vector (mock), *SNORA37*, scramble shRNA (sh-Scb), sh-SNORA37 #1, or sh-SNORA37 #2. **b** Co-IP and western blot assays indicating the interaction of CMTR1 with ELAVL1 in AGS cells stably transfected with sh-Scb, sh-SNORA37 #1, or sh-SNORA37 #2. **c** BiFC assay showing the interaction between CMTR1 and ELAVL1 (arrowheads) within HGC-27 cells co-transfected with pBiFC-VN173-CMTR1 and pBiFC-VC155-ELAVL1, and those stably transfected with sh-Scb or sh-SNORA37 #1, with nuclei staining by DAPI. **d** Western blot assay indicating the expression of CMTR1 or ELAVL1 in subcellular fractions of MKN-45 and AGS cells stably transfected with mock, *SNORA37*, sh-Scb, sh-SNORA37 #1, or sh-SNORA37 #2. **e** Western blot assay showing the levels of CMTR1 and ELAVL1 in subcellular fractions of AGS cells stably transfected with sh-Scb or sh-SNORA37 #1, and those co-transfected with mock or *CMTR1*. **f** Cross-linking RIP assay indicating the interaction of ELAVL1 with *CD44* pre-mRNA containing alternative splicing sites around exon 10 in AGS cells stably transfected with sh-Scb or sh-SNORA37 #1, and those co-transfected with mock or *CMTR1*. **g** and **h** RT-PCR (g) and western blot (h) assays showing the alternative splicing and expression of *CD44v6* and *CD44s* in AGS cells stably transfected with sh-Scb or sh-SNORA37 #1, and those co-transfected with mock, *CMTR1*, or *ELAVL1*. Data are shown as representative of three independent experiments in **a-h**

To investigate the impact of *SNORA37* on CMTR1-ELAVL1 interplay, western blot and qRT-PCR assays indicated that modulation of *SNORA37* levels did not alter the transcriptional or protein levels of *CMTR1* and *ELAVL1* (Fig. 6a and Additional file 1: Fig. S6a). In vitro binding assay indicated that *SNORA37* was not able to bind to MBP-tagged ELAVL1 protein (Additional file 1: Fig. S6b). Instead, *SNORA37*, but not its antisense transcript, promoted the binding of CMTR1 to ELAVL1 protein (Additional file 1: Fig. S6c). Co-IP, western blot, and BiFC assays revealed that stable over-expression or silencing of *SNORA37* facilitated or attenuated the interaction between CMTR1 and ELAVL1 (Additional file 1: Fig. S6d, e and Fig. 6b, c). Subcellular fractionation assay indicated the increase or decrease in nuclear retention of ELAVL1 in MKN-45 or AGS cells with stable over-expression or knockdown of *SNORA37* (Fig. 6d), which was abolished by silencing or ectopic expression of *CMTR1* (Additional file 1: Fig. S7a and Fig. 6e). In addition, there was facilitated or reduced ELAVL1 enrichment on *CD44* pre-mRNA in gastric cancer cells with stable over-expression or knockdown of *CMTR1*, while silencing or ectopic expression of *SNORA37* abolished these effects (Fig. 6f and Additional file 1: Fig. S7b, c). Moreover, the decrease or increase in *CD44v6* splicing, *CD44v6*/*CD44s* ratio, and *CD44v6* expression in *SNORA37* silencing or over-expressing gastric cancer cells were restored by transfecting expression vector or shRNA specific for *CMTR1* or *ELAVL1* (Fig. 6g, h and Additional file 1: Fig. S7d-f). These results suggested that *SNORA37* facilitated CMTR1-ELAVL1 interplay in alternative splicing of *CD44*.

### *SNORA37* promotes gastric cancer progression via facilitating CMTR1-ELAVL1 interplay

**Fig. 7:**
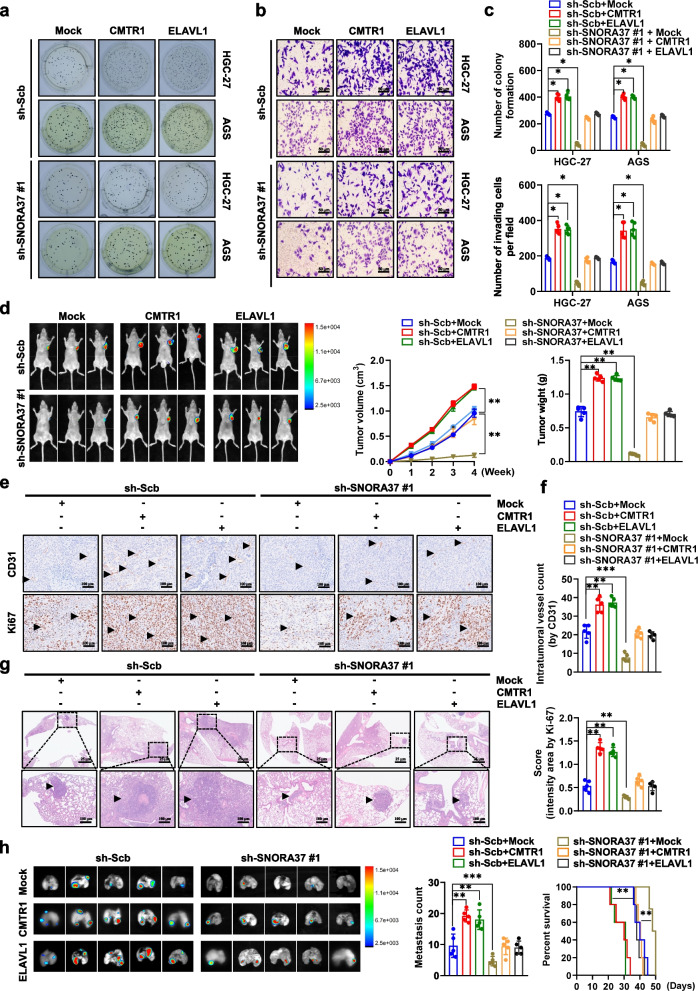
*SNORA37* promotes gastric cancer progression via facilitating CMTR1-ELAVL1 interplay. **a**-**c** Representative images (a and b) and quantification (c) of soft agar and matrigel invasion assays indicating the in vitro growth and invasion of HGC-27 and AGS cells stably transfected with scramble shRNA (sh-Scb) or sh-SNORA37 #1, and those co-transfected with *CMTR1* or *ELAVL1* (*n* = 5). **d** In vivo imaging, growth curve, and weight at the endpoints of xenograft tumors formed by subcutaneous injection of HGC-27 cells stably transfected with sh-Scb or sh-SNORA37 #1, and those co-transfected with *CMTR1* or *ELAVL1* into dorsal flanks of nude mice (*n* = 5 for each group). **e** and **f** Representative images (e) and quantification (f) of immunohistochemical staining showing the intertumoral expression of CD31 and Ki-67 (brown, arrowheads) within subcutaneous xenograft tumors of nude mice formed by HGC-27 cells stably transfected with sh-Scb or sh-SNORA37 #1, and those co-transfected with *CMTR1* or *ELAVL1* (*n* = 5 for each group). **g** and **h** Hematoxylin & eosin (HE) staining (g), representative images (h, left panel) and quantification (h, middle panel) of lung metastatic colonization (arrowheads) and Kaplan–Meier curves (h, right panel) of nude mice treated with tail vein injection of HGC-27 cells stably transfected with sh-Scb or sh-SNORA37 #1, and those co-transfected with *CMTR1* or *ELAVL1* into tail vein of nude mice (*n* = 5 for each group). One-way analysis of variance (ANOVA) was used to compare the difference in **c**, **d**, **f**, and **h**. Log-rank test was used for survival comparison in **h**. *P < 0.05, ** *P* < 0.01, *** *P* < 0.001. Data are shown as mean ± SEM (error bars) and representative of three independent experiments in **a**-**c**

To further explore the impact of *SNORA37* on CMTR1-ELAVL1 interplay during cancer progression, rescue experiments were performed. The anchorage-independent growth and invasive capacity of HGC-27 and AGS cells were enhanced or repressed by over-expression or silencing of *CMTR1* or *ELAVL1*, while *SNORA37* knockdown or over-expression counteracted these alterations (Fig. 7a-c and Additional file 1: Fig. S8a-c). Stable over-expression of *CMTR1* or *ELAVL1* into HGC-27 cells resulted in increase of growth, tumor weight, CD31-positive microvessels, and Ki-67 proliferative index of subcutaneous xenografts formed in nude mice, accompanied by up-regulation of CD44v, which were prevented by knockdown of *SNORA37* (Fig. 7d-f and Additional file 1: Fig. S8d, e). In experimental metastasis assay, athymic nude mice injected with HGC-27 cells stably transfected with *CMTR1* or *ELAVL1* into tail vein had more lung or liver metastasis and a lower survival probability, which were restored by *SNORA37* silencing (Fig. 7g, h and Additional file 1: Fig. S8f). These results suggested that *SNORA37* promoted gastric cancer progression via facilitating CMTR1-ELAVL1 interplay.

### *SNORA37*/CMTR1/ELAVL1 axis is associated with poor prognosis of gastric cancer patients

**Fig. 8:**
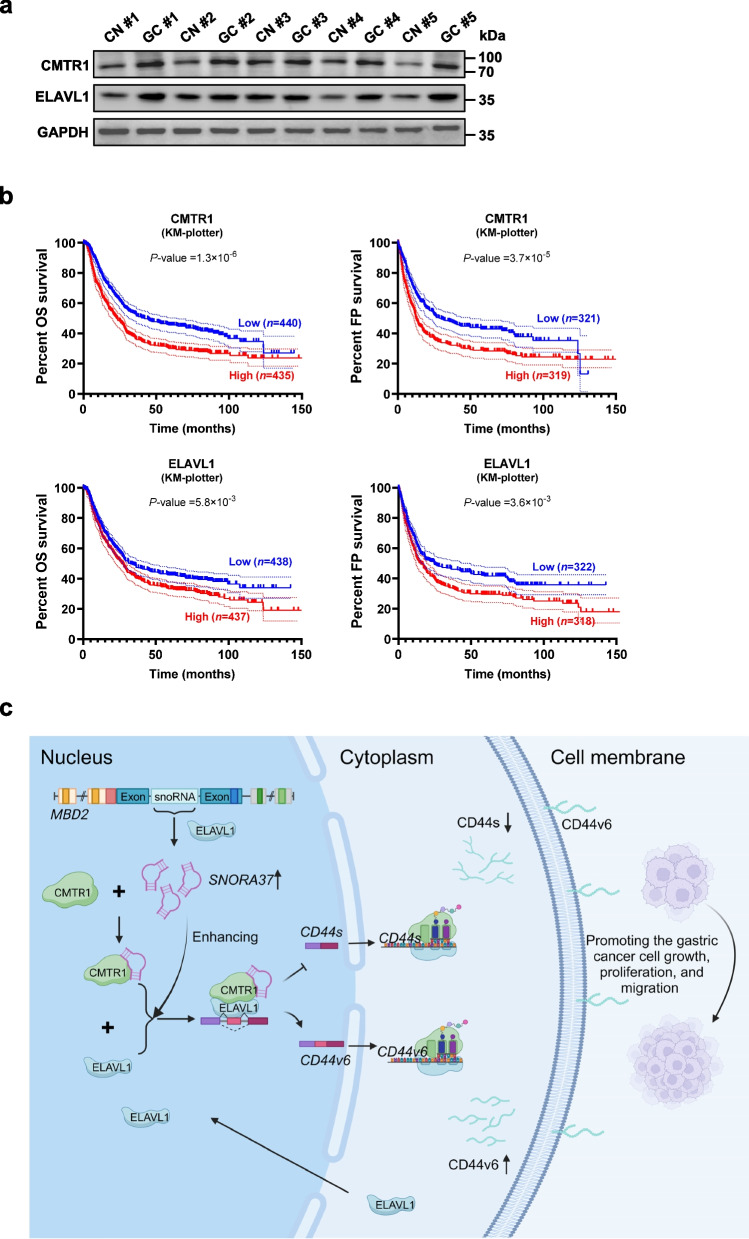
*SNORA37*/CMTR1/ELAVL1 axis is associated with poor prognosis of gastric cancer patients. **a** Western blot assay showing the expression of CMTR1 and ELAVL1 in gastric cancer (GC) and corresponding normal (CN) epithelial tissues. **b** Kaplan–Meier curves indicating overall survival (OS) and first progression (FP) survival of gastric cancer cases with low or high levels of *CMTR1* (cutoff values = 8.96 and 8.82, respectively) or *ELAVL1* (cutoff values = 8.33 and 8.18, respectively). **c** The mechanism of *SNORA37*/CMTR1/ELAVL1 axis-mediated cancer progression: As an ELAVL1-facilitated H/ACA box snoRNA derived from host gene *MBD2*, *SNORA37* directly binds to CMTR1 to promote its interaction with ELAVL1, resulting in nuclear retention and activity of ELAVL1 in regulating alternative splicing of *CD44*. *SNORA37* exerts oncogenic roles in gastric cancer progression via facilitating CMTR1-ELAVL1 interaction. Log-rank test for survival comparison in **b**. Data are shown as representative of three independent experiments in **a**

To ascertain the relationship of *SNORA37*, *CMTR1* and *ELAVL1* with gastric cancer prognosis, their expression was measured in clinical samples. Western blotting assay showed that CMTR1 and ELAVL1 were up-regulated in human gastric cancer tissues than those in paired normal epithelia (Fig. 8a). The Kaplan–Meier survival plots from KM-Plotter database revealed that high expression levels of *CMTR1* (*P-*value = 1.3 × 10^–6^ and 3.7 × 10^–5^, respectively) or *ELAVL1* (*P-*value = 5.8 × 10^–3^ and 3.6 × 10^–3^, respectively) were associated with lower OS and FP survival probabilities of gastric cancer parents (Fig. 8b). These data indicated that *SNORA37*/CMTR1/ELAVL1 axis was associated with poor prognosis of gastric cancer patients.

## Discussion

SnoRNAs are involved in 2'-O-methylation or pseudouridine modification of rRNAs and other RNA molecules within nucleolus [33], while they are also present in cytoplasm [34] or mitochondria [35], suggesting their additional biological functions. Recent evidence shows the potential role of snoRNAs in the development and progression of cancers [13, 15, 36, 37]. For example, *SNORD88C* activates the phosphorylation of mechanistic target of rapamycin kinase (mTOR)/unc-51 like autophagy activating kinase 1 (ULK1) to inhibit autophagy, thus driving lung tumor invasion and metastasis [15]. *SNORD17* deletion promotes the cytoplasmic translocation of nucleophosmin 1 (NPM1) and MYB binding protein 1a (MYBBP1A) to facilitate their interaction with mouse 3T3 cell double minute 2 (MDM2) or p300 essential for p53 stabilization, thereby suppressing tumor progression [36]. *SNORA38B* drives tumor progression by stimulating secretion of interleukin-10, which recruits regulatory T cells and reduces infiltration of CD3^+^ and CD8^+^ T cells in non-small cell lung cancer (NSCLC) [37]. However, the roles of snoRNAs in gastric cancer progression remain largely unknown. In this study, we identified several snoRNAs with altered expression in gastric cancer. *SNORA37*, an ELAVL1-facilitated H/ACA box snoRNA derived from host gene *MBD2*, was up-regulated in gastric cancer tissues and associated with poor outcomes of patients. We demonstrated that *SNORA37* exerted oncogenic roles in tumorigenesis and aggressiveness. Although previous studies implicate the potential correlation between snoRNA and host gene expression [38, 39], our results indicated no impact of *SNORA37* on *MBD2* expression. Instead, *SNORA37* was able to regulate alternative splicing via facilitating the interaction between CMTR1 and ELAVL1 (Fig. 8c). Especially, *SNORA37* promoted the inclusion of exon 10 in *CD44* or exclusion of exons 8 and 9 in *PRMT2*, leading to abundant expression of *CD44v6* or *PRMT2γ* isoform. Since previous studies show that both *CD44v6* and *PRMT2γ* play crucial roles in cancer pathogenesis [17, 18, 40], our findings indicated the oncogenic roles of *SNORA37* via modulating alternative splicing of oncogenes.

CMTR1 was initially identified as a cap methyltransferase involved in mRNA cap modifications [41]. Subsequent studies show that CMTR1 participates in tumorigenesis. For example, CMTR1 is able to recruit RNA polymerase II to transcription start site of signal transducer and activator of transcription 3 (*STAT3*) essential for cancer cell proliferation and anti-tumor immunity, while suppression of CMTR1 significantly boosts the efficacy of programmed death-1 (PD1) blockade by encouraging the infiltration of CD8^+^ T cells in tumor microenvironment [42]. *CMTR1* can contribute to anaplastic lymphoma kinase (*ALK*) gene frameshift mutations via gene fusion, which leads to clotozantinib resistance in NSCLC [43]. As a protein partner, DEAH-box helicase 15 (DHX15) enhances the cap methyltransferase activity of CMTR1 [44]. In addition, casein kinase II (CK2) facilitates the phosphorylation of CMTR1 to enhance co-transcriptional capping [45]. In this study, our results revealed the unexpected roles of *CMTR1* in regulating alternative splicing of *CD44* via facilitating nuclear retention of ELAVL1. We demonstrated that G-patch domain of CMTR1 and Hinge domain of ELAVL1 were required for their interaction, suggesting their interplay in cancer progression.

ELAVL1 predominantly functions as a RBP that regulates mRNA stability via binding to 3'-untranslated region (3'-UTR) [46]. Within the nucleus, ELAVL1 plays a role in regulating alternative splicing of eukaryotic translation initiation factor 4E-transporter (*4E-T*) to facilitate postnatal angiogenesis [8]. It can also undergo nuclear-cytoplasmic shuttling regulated by AMP-activated kinase, protein kinase C, or mitogen-activated protein kinase [47]. Additionally, *circAGO2* binds to ELAVL1 to facilitate its translocation from nucleus to cytoplasm [28]. It has been established that *ELAVL1* is up-regulated and associated with unfavorable outcomes in various cancer types, including lung cancer, ovarian cancer, pancreatic cancer, meningioma, esophageal squamous cell carcinoma, gastric cancer, and bladder cancer [28, 46, 48]. However, the potential role of ELAVL1 as a splicing factor in tumorigenesis remains to be determined. In this study, our data showed that nuclear ELAVL1 was essential for alternative splicing of cancer-related genes such as *CD44*. CMTR1 was able to enhance the nuclear retention of ELAVL1 via interacting with its hinge region. Previous studies have suggested that hinge region of ELAVL1, locating between RRM2 and RRM3, contains a nucleocytoplasmic shuttle sequence (HNS, spanning residues 205–237) that binds to transportin 2 (TRN2), facilitating the export of ELAVL1 to cytoplasm [47, 49, 50]. Therefore, we speculate that competitive binding of CMTR1 and TRN2 might regulate nuclear retention of ELAVL1 in cancer cells, which warrants further investigation. We discovered that blocking the interaction of CMTR1 with ELAVL1 via *SNORA37* knockdown was able to repress tumorigenesis and aggressiveness, indicating the value of *SNORA37*/CMTR1/ELAVL1 axis as a therapeutic target for cancers.

## Conclusion

In conclusion, we demonstrate, for the first time, that *SNORA37* is up-regulated in gastric cancer tissues and associated with poor outcomes of patients. As an ELAVL1-facilitated snoRNA, *SNORA37* binds with CMTR1 to regulate nuclear retention of ELAVL1 by enhancing their interaction, leading to increased activity of ELAVL1 in regulating inclusion of *CD44* exon 10, which ultimately promotes tumorigenesis and aggressiveness. Meanwhile, the roles of *SNORA37* in regulating structure of CMTR1 crucial for its binding to ELAVL1 need additional studies. This study is helpful for expanding our understanding of pre-mRNA alternative splicing regulation by snoRNA and protein partners, and suggests that targeting *SNORA37*/CMTR1/ELAVL1 feedback loop is a potential therapeutic strategy for human cancers, while its potential role in tumor microenvironment warrants further investigation.

## Supplementary Information

Additional file 1: Figure S1. Expression profiles of snoRNAs in gastric cancer tissues. Figure S2. Oncogenic roles of *SNORA37* in gastric cancer. Figure S3. Expression profiles and alternative splicing of *CD44* and* PRMT2* in gastric cancer. Figure S4. *SNORA37 *interacts with CMTR1. Figure S5. CMTR1 facilitates nuclear retention of oncogenic ELAVL1 in gastric cancer cells. Figure S6. *SNORA37* enhances the interaction of CMTR1 with ELAVL1. Figure S7. *SNORA37* facilitates alternative splicing of *CD44* via CMTR1 and ELAVL1. Figure S8. *SNORA37* facilitates gastric cancer progression via enhancing interaction of CMTR1 with ELAVL1. Table S1. Primer sets used for RT-PCR, RIP, and probe. Table S2. Primer sets used for constructs. Table S3. Oligonucleotide sets for short hairpin RNAs. Table S4. Differentially expressed snoRNAs in RNA-seq assay. Table S5. Alteration of splicing events in MKN-45 cells upon *SNORA37* over-expression. Table S6. Alteration of splicing events in gastric tissues. Table S7. Mass spectrometry analysis of proteins pulled down by *SNORA37*.

## Data Availability

RNA-seq results have been deposited at GEO database (https://www.ncbi.nlm.nih.gov/geo, accession number GSE285296 and GSE285402). Public snoRNA or gene transcript expression datasets are available from The Cancer Genome Atlas (TCGA, https://cancergenome.nih.gov), Tumor-Normal-Metastatic-plot (TNM-plot, https://tnmplot.com), TSVdb (http://www.tsvdb.com), or Genotype-Tissue Expression (GTEx, https://www.gtexportal.org) database. By searching the name of relevant snoRNA or gene, the expression data are available and visualized by using GraphPad 8.0 software (GraphPad Software, San Diego, CA). All CMTR1-interacting protein can be obtained by searching the BioGRID database (https://thebiogrid.org) with the keyword "CMTR1". As a website for Gene Ontology pathway analysis, Metascape program (https://metascape.org) is applied by pasting a list of proteins and selecting the parameter “Express Analysis”. The ELAVL1 binding sites within input pre-mRNA sequence were analyzed by RBPmap (http://rbpmap.technion.ac.il/) program, using mandatory motif selection mode. Kaplan–Meier survival plots are obtained from KM-Plotter database (http://kmplot.com). The patients are divided into high or low groups by medium gene expression levels as cutoffs. The event status (dead or alive) and survival time of each patient in low or high groups are collected and subjected to Kaplan–Meier curve generation or statistical analysis using GraphPad 8.0 software. The data supporting the conclusions of this article are presented within the article and its Additional files.

## Abbreviations

3'-UTR: 3'-Untranslated region
4E-T: Eukaryotic translation initiation factor 4E-transporter
5-HT2CR: 5-hydroxytryptamine receptor 2C
A3SS: Alternative 3' splice sites
A5SS: Alternative 5' splice sites
ALK: Anaplastic lymphoma kinase
ANOVA: Analysis of variance
AREs: AU-rich elements
AS: Alternative splicing
BiFC: Bimolecular fluorescence complementation
CD44s: CD44 standard isoform
CD44v: CD44 variant isoform
CK2: Casein kinase II
CMTR1: Cap-specific mRNA (nucleoside-2'-O-)-methyltransferase 1
Co-IP: Co-immunoprecipitation
DAPI: 4',6-Diamidino-2-phenylindole
DHX15: DEAH-box helicase 15
ELAVL1: ELAV like RNA binding protein 1
FISH: Fluorescence in situ hybridization
FP: First progression
GAPDH: Glyceraldehyde-3-phosphate dehydrogenase
GST: Glutathione S-transferase
HA: Hemagglutinin
HE: Hematoxylin and eosin
HNRNPC: Heterogeneous nuclear ribonucleoprotein C
HNRNPs: Heterogeneous nuclear ribonucleoproteins
HNRNPU: Heterogeneous nuclear ribonucleoprotein U
IGF2BP2: Insulin-like growth factor 2 mRNA-binding protein 2
MBD2: Methyl-CpG binding domain protein 2
MBP: Myelin basic protein
MDM2: Mouse 3T3 cell double minute 2
Mock: Empty vector
MS: Mass spectrometry
mTOR: Mechanistic target of rapamycin kinase
MTT: 2-(4,5-Dimethyltriazol-2-yl)-2,5-diphenyl tetrazolium bromide
MXE: Mutually exclusive exons
MYBBP1A: MYB binding protein 1a
NPM1: Nucleophosmin 1
NSCLC: Non-small cell lung cancer
NVL: Nuclear valosin-containing protein-like
OS: Overall survival
PARPBP: Poly(ADP-ribose) polymerase 1 binding protein
pre-mRNA: Precursor messenger RNA
PRMT2: Protein arginine methyltransferase 2
PSI: Precent spliced-in
PTBP1: Polypyrimidine tract-binding protein 1
qRT-PCR: Quantitative RT-PCR
RAP1B: RAS-related protein 1B
RBP: RNA-binding protein
RI: Retained intron
RIP: RNA immunoprecipitation
RPKM: Reads per kilobase of transcript per million mapped reads
scaRNAs: Small Cajal body-associated RNAs
SD: Standard deviation
SE: Skipping exon
SF: Splicing factor
SF3B1: Splicing factor 3B subunit 1
shRNA: Short hairpin RNA
sh-Scb: Scramble shRNA
snoRNA: Small nucleolar RNA
snRNAs: Small nuclear RNAs
SR: Serine/arginine-rich
SRSF1: Serine/arginine-rich splicing factor 1
STAT3: Signal transducer and activator of transcription 3
U2AF2: U2 small nuclear RNA auxiliary factor 2
ULK1: Unc-51 like autophagy activating kinase 1
UREs: U-rich elements
