# Supplementary material for: SNORA37/CMTR1/ELAVL1 feedback loop drives gastric cancer progression via facilitating CD44 alternative splicing
Source: J Exp Clin Cancer Res. 2025 Jan 16;44:15. doi: 10.1186/s13046-025-03278-x (PMC11737211; doi:10.1186/s13046-025-03278-x)
Supplement: Supplementary file 1 — Additional file 1: Figure S1. Expression profiles of snoRNAs in gastric cancer tissues. Figure S2. Oncogenic roles of SNORA37 in gastric cancer. Figure S3. Expression profiles and alternative splicing of CD44 and PRMT2 in gastric cancer. Figure S4. SNORA37 interacts with CMTR1. Figure S5. CMTR1 facilitates nuclear retention of oncogenic ELAVL1 in gastric cancer cells. Figure S6. SNORA37 enhances the interaction of CMTR1 with ELAVL1. Figure S7. SNORA37 facilitates alternative splicing of CD44 via CMTR1 and ELAVL1. Figure S8. SNORA37 facilitates gastric cancer progression via enhancing interaction of CMTR1 with ELAVL1. Table S1. Primer sets used for RT-PCR, RIP, and probe. Table S2. Primer sets used for constructs. Table S3. Oligonucleotide sets for short hairpin RNAs. Table S4. Differentially expressed snoRNAs in RNA-seq assay. Table S5. Alteration of splicing events in MKN-45 cells upon SNORA37 over-expression. Table S6. Alteration of splicing events in gastric tissues. Table S7. Mass spectrometry analysis of proteins pulled down by SNORA37. [file 13046_2025_3278_MOESM1_ESM.pdf]

Supporting Information

***SNORA37*/CMTR1/ELAVL1 feedback loop drives gastric cancer progression via facilitating *CD44* alternative splicing**

Bao et al.

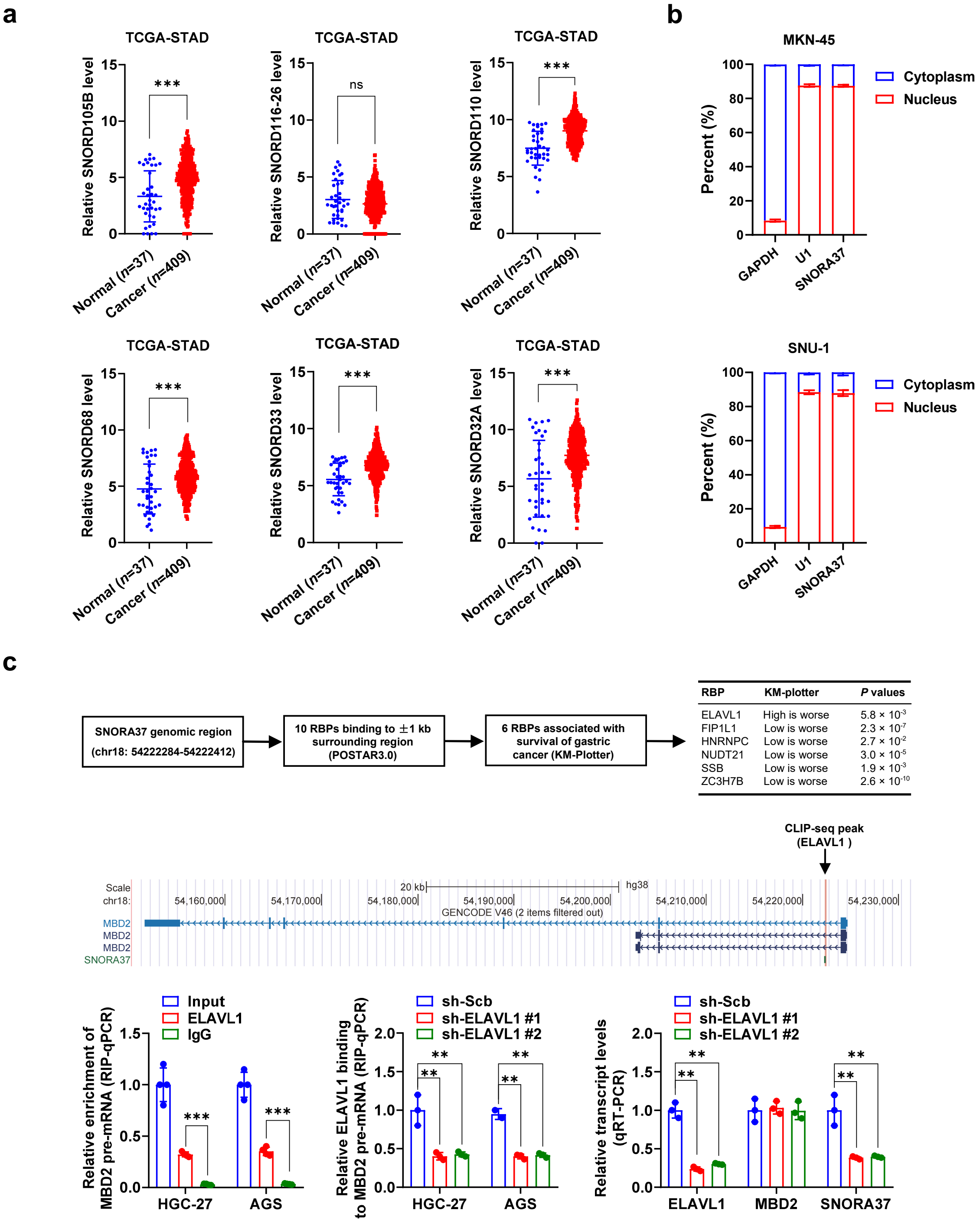

Figure S1. Expression profiles of snoRNAs in gastric cancer tissues. a, The levels of *SNORD105B*, *SNORA116-26*, *SNORD110*, *SNORD68*, *SNORD33*, and *SNORD32A* in normal gastric epithelia (*n*=37) and gastric cancer tissues (*n*=409) derived from TCGA datasets. **b**, Real-time qRT-PCR indicating the distribution of *SNORA37*, *GAPDH*, and *U1* in cytoplasmic and nuclear fractions of MKN-45 and SNU-1 (*n*=4). **c**, Identification of potential RBPs regulating *SNORA37* expression and their association with survival of gastric cancer patients by comprehensive analysis of datasets derived from POSTAR 3.0 (http://111.198.139.65/) and KM-Plotter (http://kmplot.com) databases (upper panel). CLIP-seq dataset (middle panel) derived from ENCORI (https://rnasysu.com/encori/) revealing the enrichment of ELAVL1 on pre-mRNA of *SNORA37* host gene *MBD2*. RIP and qRT-PCR (lower left and middle panels) assays showing the binding of ELAVL1 (normalized to input, *n*=4) to *MBD2* pre-mRNA in HGC-27 and AGS cells, and those stably transfected with scramble shRNA (sh-Scb), sh-ELAVL1 #1, or sh-ELAVL1 #2 (*n*=3). Real-time qRT-PCR (lower right panel) indicating the levels (normalized to *β-actin*, *n*=3) of *ELAVL1*, *MBD2*, or *SNORA37* in HGC-27 cells stably transfected with sh-Scb, sh-ELAVL1 #1, or sh-ELAVL1 #2. **$P<0.01$, ***$P<0.001$ vs. normal, IgG, or sh-Scb. Non-parametric Mann-Whitney U test compared the difference in **a**. One-way analysis of variance (ANOVA) compared the difference in **c**. Data are shown as mean ± s.e.m. (error bars) or representative of three independent experiments in **a**-**c**.

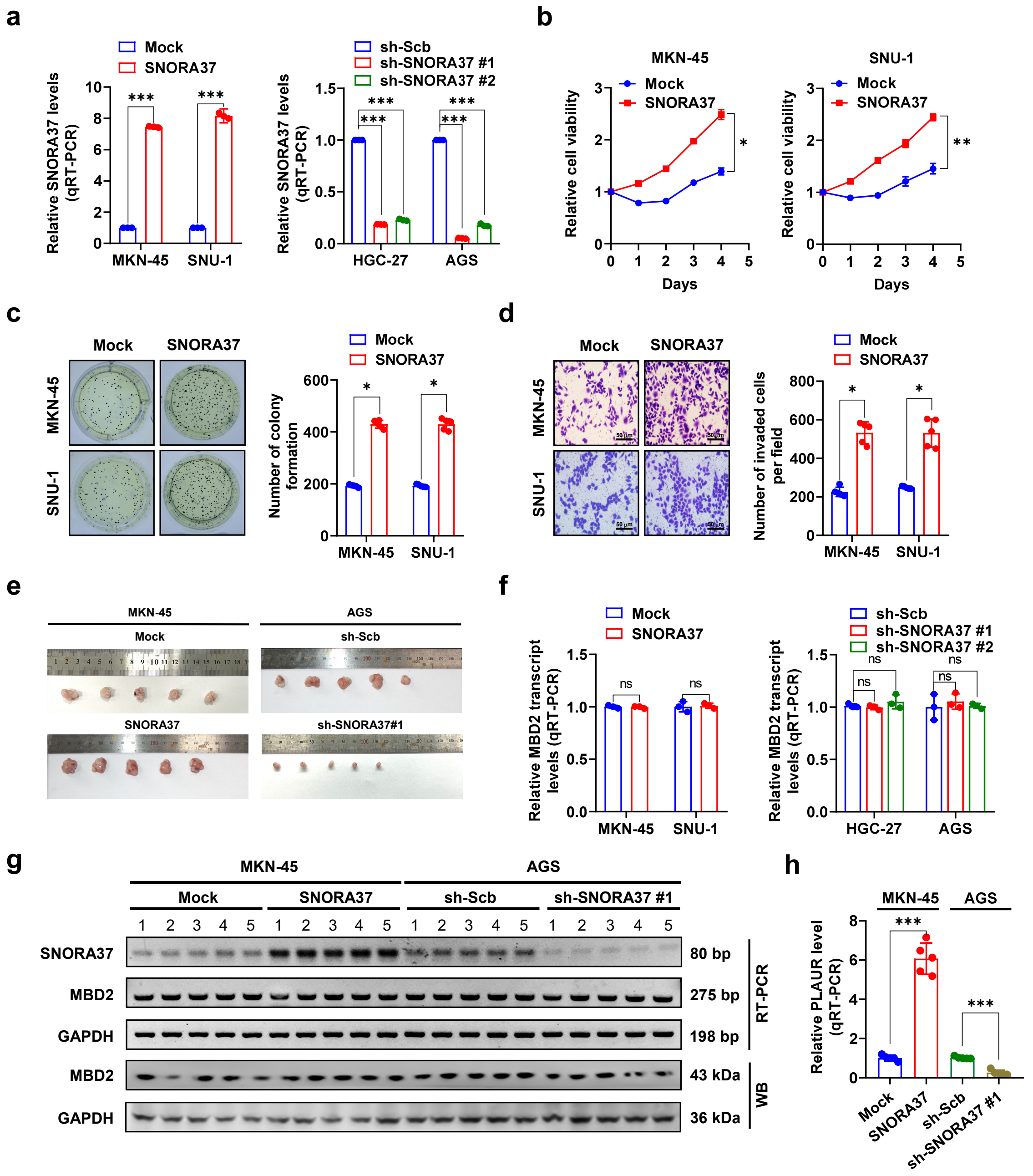

Figure S2. Oncogenic roles of *SNORA37* in gastric cancer. **a**, Real-time qRT-PCR assay indicating the expression of *SNORA37* (normalized to *GAPDH*) in MKN-45, SNU-1, HGC-27, and AGS cells stably transfected with empty vector (mock), *SNORA37*, scramble shRNA (sh-Scb), sh-SNORA37 #1, or sh-SNORA37 #2 (*n*=3). **b**, MTT colorimetric assay showing the relative viability of MKN-45 and SNU-1 cells stably transfected with mock or *SNORA37* (*n*=5). **c** and **d**, Representative images (left panel) and quantification (right panel) of soft agar (c) and matrigel invasion (d) assays indicating the *in vitro* growth and invasion of MKN-45 and SNU-1 cells stably transfected with mock or *SNORA37* (*n*=5). **e**, Size of xenograft tumors formed by subcutaneous injection of MKN-45 cells stably transfected with mock or *SNORA37*, or AGS cells stably transfected with sh-Scb or sh-SNORA37 #1 into dorsal flanks of nude mice (*n* = 5 for each group). **f**, Real-time qRT-PCR assay indicating the relative *MBD2* levels (normalized to *GAPDH*) in MKN-45, SNU-1, HGC-27, and AGS cells stably transfected with mock, *SNORA37*, sh-Scb, sh-SNORA37 #1, or sh-SNORA37 #2 (*n*=3). **g**, Real-time qRT-PCR and western blot assays indicating the levels of *SNORA37* and *MBD2* within subcutaneous xenograft tumors in nude mice formed by MKN-45 and AGS cells stably transfected with mock, *SNORA37*, sh-Scb, or sh-SNORA37 #1 (*n* = 5 for each group). **h**, Real-time qRT-PCR assay showing the relative levels (normalized to *GAPDH*) of *PLAUR* within liver metastatic foci formed by intravenous injection of MKN-45 and AGS cells stably transfected with mock, *SNORA37*, sh-Scb, or sh-SNORA37 #1 (*n* = 5 for each group). Student's *t*-test and one-way analysis of variance (ANOVA) were used to compared the difference in **a-d**, **f** and **h**. *P<0.05, ** P <0.01, *** P<0.001. Data are shown as mean ± s.e.m. (error bars) and representative of three independent experiments in **a-d**, **f** and **h**.

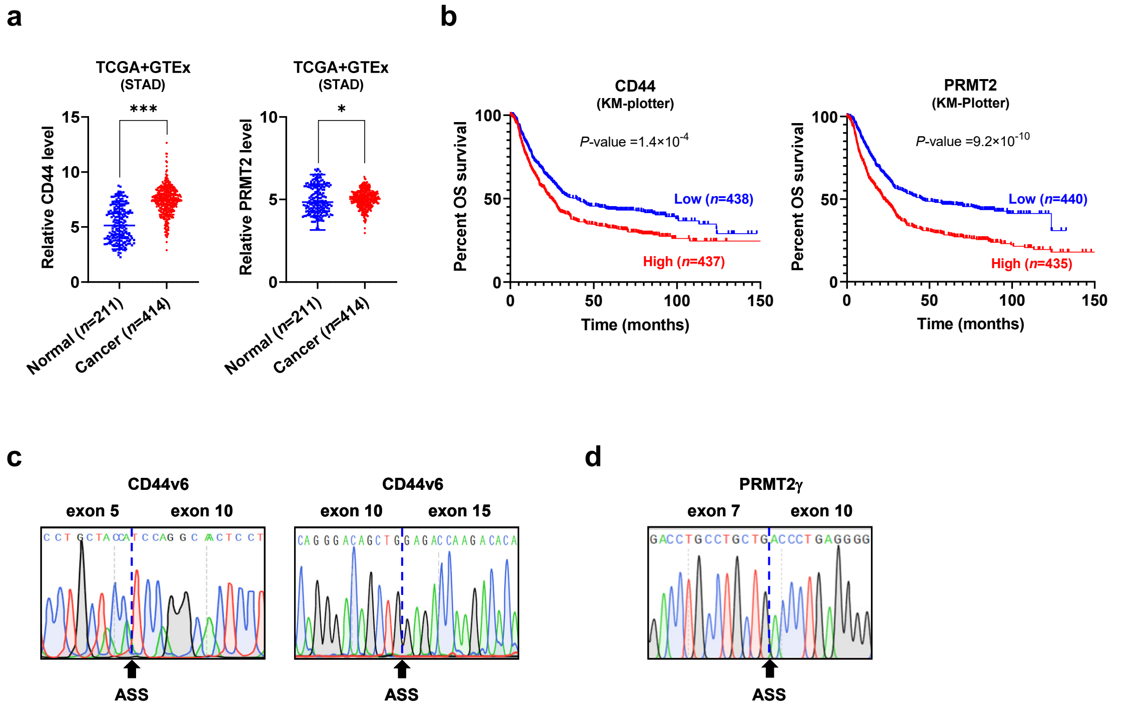

Figure S3. Expression profiles and alternative splicing of *CD44* and *PRMT2* in gastric cancer. a, Relative levels of *CD44* and *PRMT2* in normal gastric epithelia (n=211) and tumor tissues (n=414) of gastric cancer cases derived from TCGA (https://cancergenome.nih.gov) and Genotype-Tissue Expression (GTEx) database (https://www.gtexportal.org). **b**, Kaplan-Meier curves indicating overall survival (OS) of gastric cancer patients with low or high expression of *CD44* (cutoff value = 8.73) or *PRMT2* (cutoff value = 4.81) derived from KM-Plotter database (http://kmplot.com). **c**, Sanger sequencing indicating alternative splicing sites of *CD44* (C) and *PRMT2* (D) in gastric cancer AGS cells. Non-parametric Mann-Whitney U test compared the difference in **a**. Log-rank test was used for survival comparison in **b**. *P<0.05, *** P<0.001. ns, non-significant.

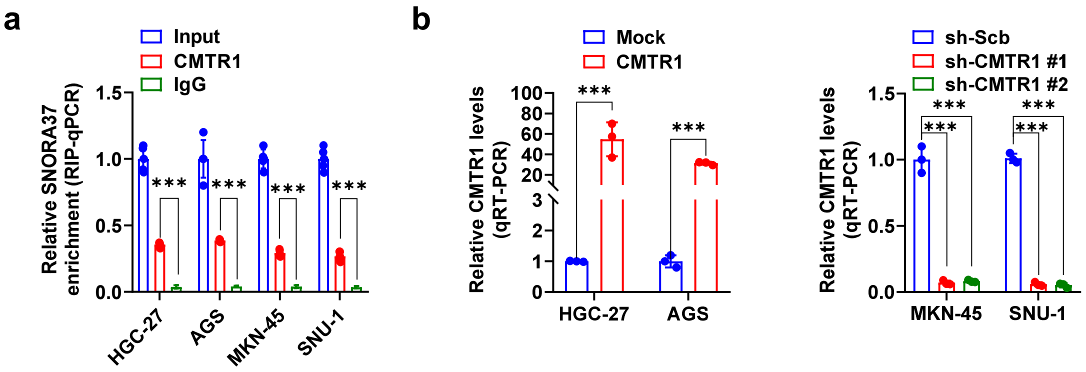

Figure S4. ***SNORA37* interacts with CMTR1. a**, RIP and real-time qRT-PCR assays showing the relative *SNORA37* levels (normalized to input, n=5) immunoprecipitated by CMTR1 antibody or IgG from HGC-27, AGS, MKN-45, or SNU-1 cell lysates. **b**, Real-time qRT-PCR assay indicating the expression (normalized to *GAPDH*, n=3) of *CMTR1* in HGC-27, AGS, MKN-45, or SNU-1 cells stably transfected with empty vector (mock), *CMTR1*, scramble shRNA (sh-Scb), sh-CMTR1 #1, or sh-CMTR1 #2. Student's t-test or one-way analysis of variance (ANOVA) were used to compare the difference in **a** and **b**. *** P<0.001. Data are shown as mean ± s.e.m. (error bars) or representative of three independent experiments in **a** and **b**.

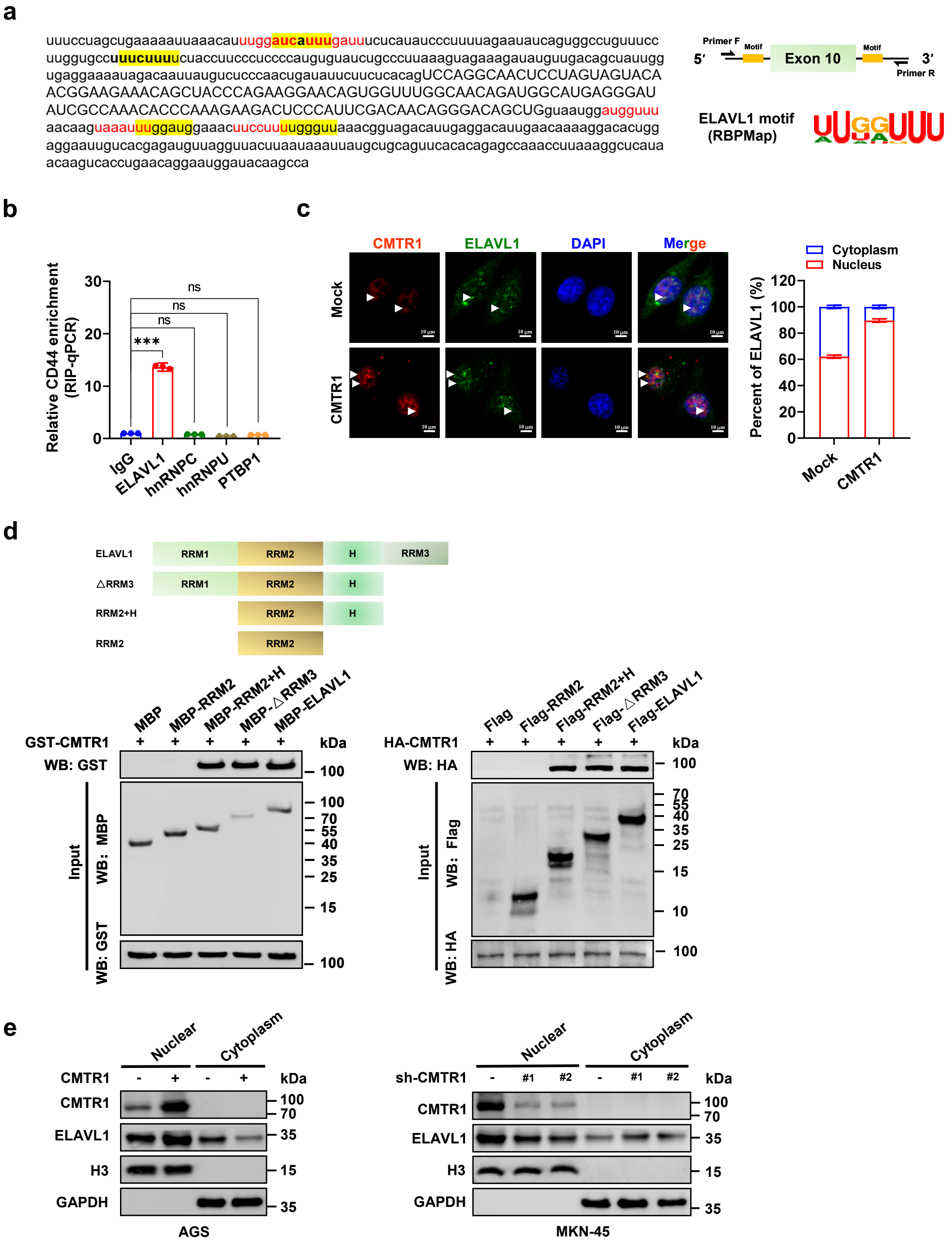

Figure S5. CMTR1 facilitates nuclear retention of oncogenic ELAVL1 in gastric cancer cells. a, RBPmap (http://rbpmap.technion.ac.il/) predicting the binding sites of ELAVL1 within intronic regions surrounding exon 10 of *CD44*. **b**, RIP and real-time qRT-PCR assays (normalized to input, *n*=3) showing the interaction of ELAVL1, HNRNPC, HNRNPU, and PTBP1 with *CD44* pre-mRNA containing alternative splicing sites around exon 10. **c**, Representative images (left panel) and quantification (right panel) of immunofluorescence assay showing the co-localization of CMTR1 and ELAVL1 (arrowheads) in HGC-27 cells stably transfected with empty vector (mock) or *CMTR1*, with nuclei staining by DAPI. **d**, *In vitro* binding (lower left panel), co-IP (lower right panel) and western blot assays showing the interaction between GST-tagged CMTR1 protein and MBP-tagged ELAVL1 truncations (upper panel), and that in AGS cells transfected with HA-tagged *CMTR1* and full-length or truncations of Flag-tagged *ELAVL1* as indicated. **e**, Western blot assay indicating the expression of CMTR1 and ELAVL1 in subcellular fractions of AGS and MKN-45 cells stably transfected with mock, *CMTR1*, scramble shRNA (sh-Scb), sh-CMTR1 #1, or sh-CMTR1 #2. GAPDH and histone H3 served as controls. One-way analysis of variance (ANOVA) was used to compare the difference in **b**. *** *P*<0.001. ns, non-significant. Data are shown as mean ± s.e.m. (error bars) or representative of three independent experiments in **b**-**e**.

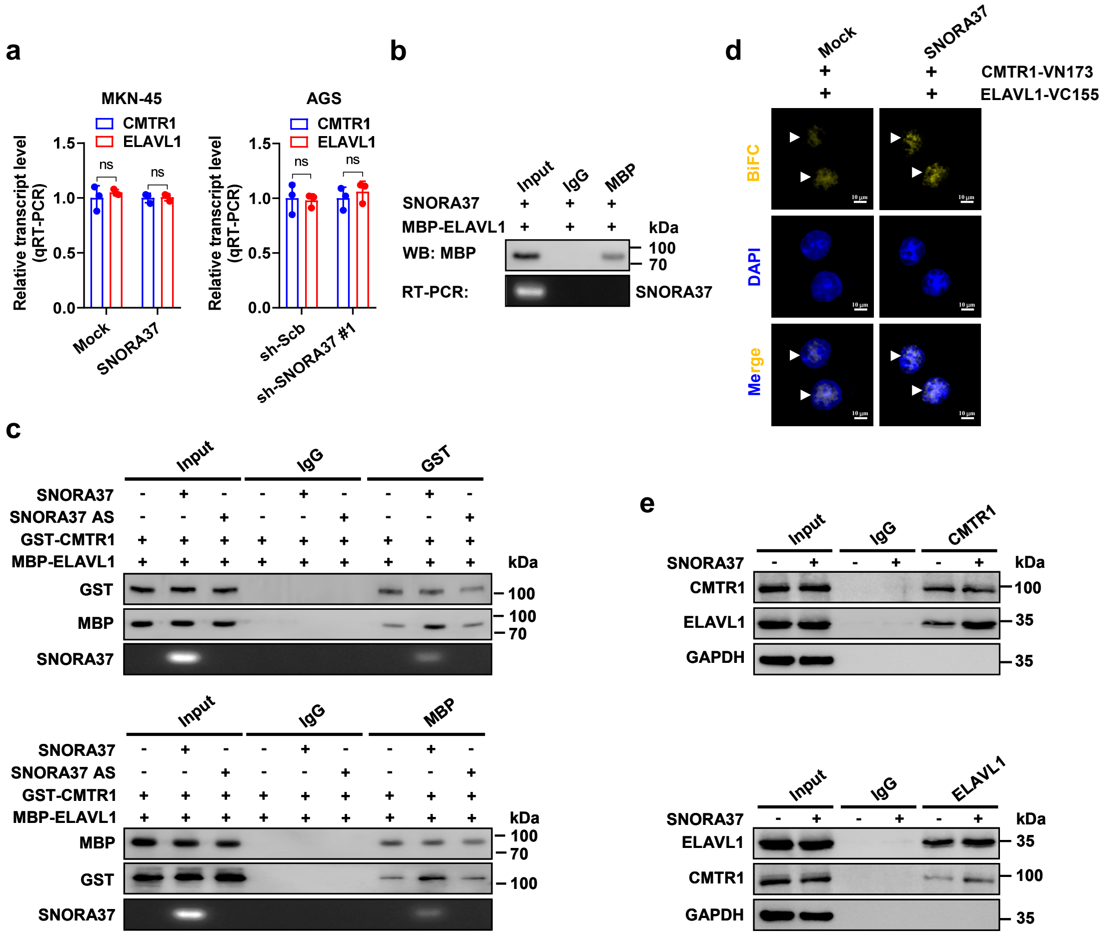

Figure S6. ***SNORA37*** **enhances the interaction of CMTR1 with ELAVL1. a**, Real-time qRT-PCR assay indicating the levels (normalized to *GAPDH*, n=3) of *CMTR1* and *ELAVL1* in MKN-45 and AGS cells stably transfected with empty vector (mock), *SNORA37*, scramble shRNA (sh-Scb), or sh-SNORA37 #1. **b** and **c**, *In vitro* binding assay revealing the interaction of *SNORA37* to MBP-tagged ELAVL1 protein (b) and that of *SNORA37* or its antisense (AS) transcript to MBP-tagged ELAVL1 and GST-tagged CMTR1 proteins (c). **d**, BiFC assay showing the interaction between CMTR1 and ELAVL1 (arrowheads) within MKN-45 cells co-transfected with pBiFC-VN173-CMTR1 and pBiFC-VC155- ELAVL1, and those stably transfected with mock or *SNORA37*, with nuclei staining by DAPI. **e**, Co-IP and western blot assays showing the interaction of CMTR1 with ELAVL1 in MKN-45 cells stably transfected with mock or *SNORA37*. Student's t-test compared the difference in **a**. ns, non-significant. Data are shown as mean ± s.e.m. (error bars) or representative of three independent experiments in **a**-**e**.

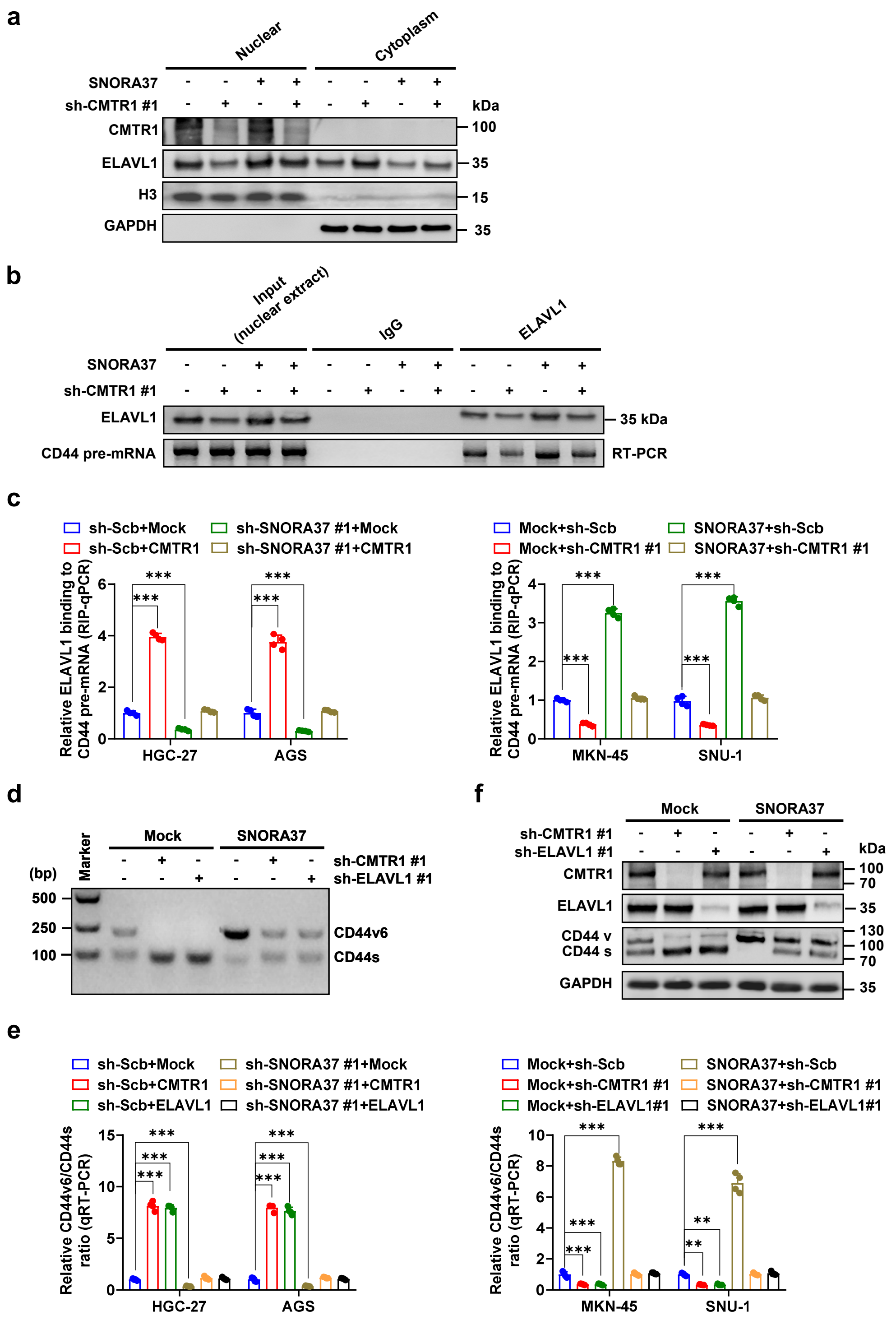

Figure S7. ***SNORA37* facilitates alternative splicing of *CD44* via CMTR1 and ELAVL1.ss a**, Western blot assay showing the levels of CMTR1 and ELAVL1 in subcellular fractions of MKN-45 cells stably transfected with empty vector (mock) or *SNORA37*, and those co-transfected with scramble shRNA (sh-Scb) or sh-CMTR1 #1. **b**, RIP assay indicating the interaction of ELAVL1 with *CD44* pre-mRNA containing alternative splicing sites around exon 10 in MKN-45 cells stably transfected with mock or *SNORA37*, and those co-transfected with sh-Scb or sh-CMTR1 #1. **c**, RIP and qRT-PCR assays showing the binding of ELAVL1 (normalized to input, n=4) to *CD44* pre-mRNA in gastric cancer cells stably transfected with sh-Scb, sh-SNORA37 #1, mock, or *SNORA37*, and those co-transfected with *CMTR1* or sh-CMTR1 #1 (*n*=4). **d**, RT-PCR assay indicating the alternative splicing and expression of *CD44v6* and *CD44s* in MKN-45 cells stably transfected with mock or *SNORA37*, and those co-transfected with sh-Scb or sh-CMTR1 #1. **e**, Real-time qRT-PCR assay showing the *CD44v6*/*CD44s* ratio in gastric cancer cells stably transfected with sh-Scb, sh-SNORA37 #1, mock, or *SNORA37*, and those co-transfected with *CMTR1*, *ELAV1*, sh-CMTR1 #1, or sh-ELAVL1 #1 (*n*=4). **f**, Western blot assay indicating the alternative splicing and expression of *CD44v6* and *CD44s* in MKN-45 cells stably transfected with mock or *SNORA37*, and those co-transfected with sh-Scb or sh-CMTR1 #1. One-way analysis of variance (ANOVA) were used to compare the difference in **c** and **e**. ** *P*<0.01, *** *P*<0.001. Data are shown as mean ± s.e.m. (error bars) or representative of three independent experiments in **a-f**.

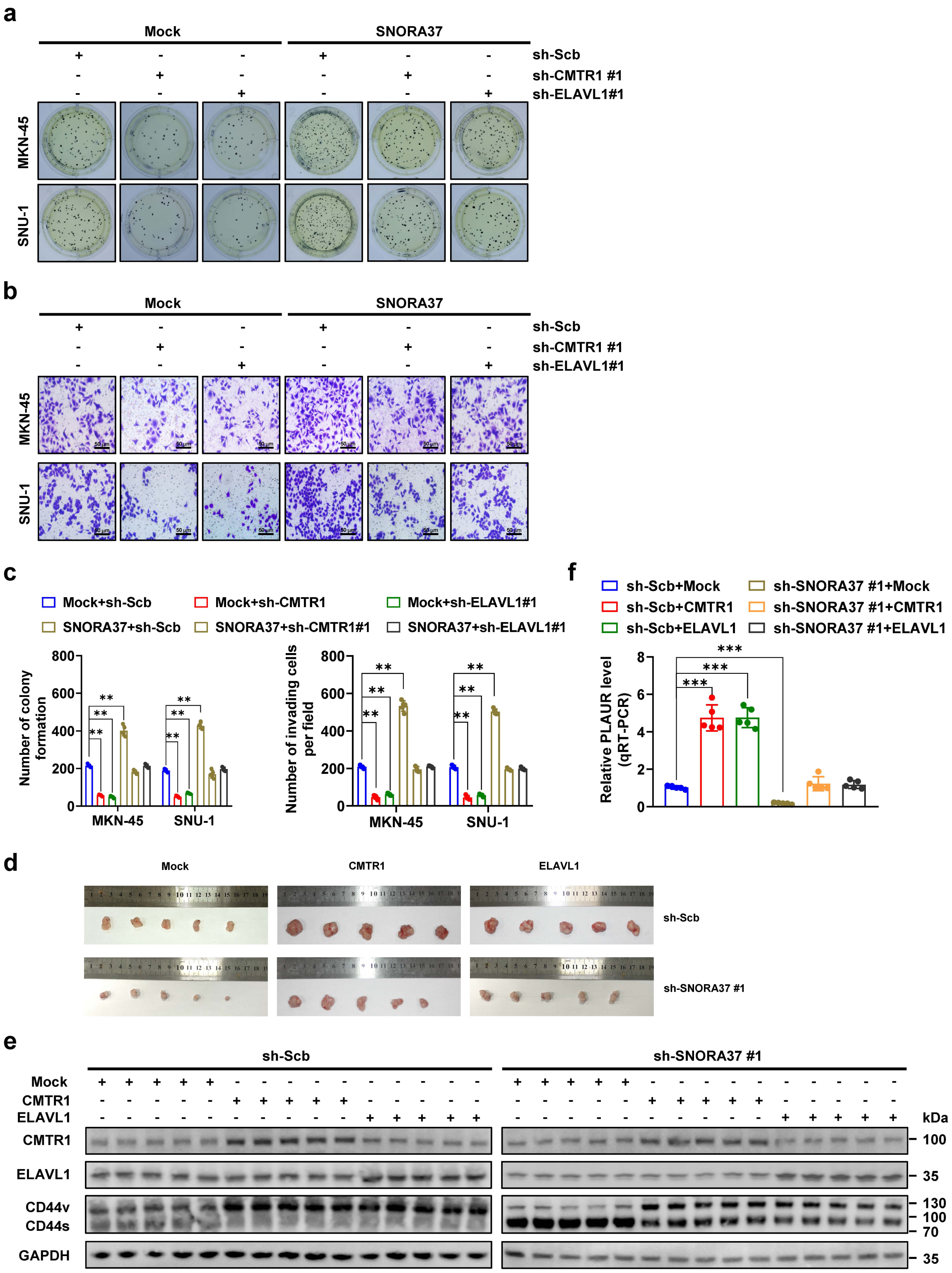

Figure S8. ***SNORA37* facilitates gastric cancer progression via enhancing interaction of CMTR1 with ELAVL1. a-c**, Representative images (a and b) and quantification (c) of soft agar and matrigel invasion assays indicating *in vitro* growth and invasion of MKN-45 and SNU-1 cells stably transfected with empty vector (mock) or *SNORA37*, and those co-transfected with scramble shRNA (sh-Scb), sh-CMTR1 #1, or sh-ELAVL1 #1 (n = 5). **d**, Size of xenograft tumors formed by subcutaneous injection of HGC-27 cells stably transfected with sh-Scb or sh-SNORA37 #1, and those co-transfected with *CMTR1* or *ELAVL1* (n = 5 for each group). **e**, Western blot assay indicating the relative levels of CMTR1, ELAVL1, CD44v or CD44s within subcutaneous xenograft tumors of nude mice formed by HGC-27 cells stably transfected with sh-Scb or sh-SNORA37 #1, and those co-transfected with *CMTR1* or *ELAVL1* (n=5 for each group). **f**, Real-time qRT-PCR assay indicating the relative levels (normalized to *GAPDH*) of *PLAUR* within liver metastatic foci formed by intravenous injection of HGC-27 cells stably transfected with sh-Scb or sh-SNORA37 #1, and those co-transfected with *CMTR1* or *ELAVL1* (n = 5 for each group). One-way analysis of variance (ANOVA) was used to compare the difference in **c** and **f**. **P<0.01, *** P<0.001. Data are shown as mean ± s.e.m. (error bars) or representative of three independent experiments in **a**-**f**.

Table S1 Primer sets used for RT-PCR, RIP, and probe

Primer set	Primers	Sequence	Product (bp)	Application
SNORD116-26	Forward	5’-CTATAAAAAAAATGGATCTCATCGG-3’	76	RT-PCR
	Reverse	5’-AGCTCACAGAAGTGGCACAGAAATG-3’		
SNORD110	Forward	5’-GACTTGCGAATCAAATCTGTCAATC-3’	63	RT-PCR
	Reverse	5’-GCTCAGAGACATGGAGACATCAGTG-3’		
SNORD105B	Forward	5’-CTCCACATGCGGCTGATGAC-3’	76	RT-PCR
	Reverse	5’-GTGCGTCAGGGCGTTTACTT-3’		
SNORD68	Forward	5’-ATTCTCCGGAATCGCTGTACG-3’	54	RT-PCR
	Reverse	5’-TGGAAAAGGGTTCAAATGTGC-3’		
SNORD33 (chr19)	Forward	5’-GGTGATGAGAACTTCTCCCACTC -3’	72	RT-PCR
	Reverse	5’-CAGATGGTAGTGCATGTGGAGTC-3’		
SNORD32A	Forward	5’-AGGTCAGTGATGAGCAACATTC-3’	54	RT-PCR
	Reverse	5’-CTCATGGCCGTGAGACTCAAAC-3’		
SNORA37	Forward	5’-TGAGCACTTTCACAGGTCCTCC-3’	80	RT-PCR, RIP
	Reverse	5’-ATGCCAACAAAGGTTTTCTCTC-3’		
SNORD87	Forward	5’-TGATGACTTAAATTACTTTTTGCCG-3’	58	RT-PCR
	Reverse	5’-TTATTTCTTCAAAGACAACCTCAGC-3’		
MBD2	Forward	5’-AAGTTCAGAAGCAAGCCTCAGTT-3’	275	RT-PCR
	Reverse	5’-TGTTCATTCATTCGTTGTGGGT-3’		
MBD2	Forward	5’-ATGATTGTGCCTGGGCAATA-3’	150	RIP
	Reverse	5’-TGAGGGAGGACCTGTGAAAG-3’		
CD44 (exon 5-exon 15)	Forward	5’-CAGACGAAGACAGTCCCTGG-3’	76/205	RT-PCR
	Reverse	5’-GGGTGGAATGTGTCTTGGTC-3’		
CD44 pre-mRNA	Forward	5’-TTTCCTAGCTGAAAAATTAAACATT-3’	522	RIP
	Reverse	5’-TATCCATTCCTGTTCAGGTGACTTG-3’		
CD44v6	Forward	5’-CAGACGAAGACAGTCCCTGG-3’	88	RIP
	Reverse	5’-CTAAACCAGGCCGTGAAGC-3’		
PRMT2 (exon 7-exon 10)	Forward	5’-GCAGAACGGCTTTGCTGAC-3’	183/489	RT-PCR
	Reverse	5’-CTAAACCAGGCCGTGAAGC-3’		
CMTR1	Forward	5’-TACCAGCCGTCCTGCCAACT-3’	268	RT-PCR
	Reverse	5’-TGTCGTGTCTTGAACAAAGGCAT-3’		
ELAVL1	Forward	5'-ATGTTCTCTCGGTTTGGGCG-3'	198	RT-PCR
	Reverse	5'-GTTCTGGTTGGGGTTGGCTG-3'		
PLAUR	Forward	5’-CAGCCTTACCGAGGTTGTGT-3’	231	RT-PCR
	Reverse	5’-TCATCCTTTGGACGCCCTTC-3’		
GAPDH	Forward	5'-GGGCTGCTTTTAACTCTGGT-3'	198	RT-PCR
	Reverse	5'-TGATTTTGGAGGGATCTCGC-3'		
T7+SNORA37 Sense	Forward	5’-TAATACGACTCACTATAGGGTGAGCACTTTCACAGGTCCTCC-3’		probe
	Reverse	5’-AATTGTCCCATTGAATGACAGCT-3’		
T7+SNORA37 Antisense	Forward	5’-TAATACGACTCACTATAGGGAATTGTCCCATTGAATGACAGCT-3’		probe
	Reverse	5’-TGAGCACTTTCACAGGTCCTCC-3’		

SNORD116-26, small nucleolar RNA, C/D box 116-26; SNORD110, small nucleolar RNA, C/D box 110; SNORD105B, small nucleolar RNA, C/D box 105B; SNORD68, small nucleolar RNA, C/D box 68; SNORD33, small nucleolar RNA, C/D box 33; SNORD32A, small nucleolar RNA, C/D box 32A; SNORA37, small nucleolar RNA, H/ACA box 37; SNORD87, small nucleolar RNA, C/D box 87; MBD2, methyl-CpG binding domain protein 2; PRMT2, protein arginine methyltransferase 2; CMTR1, cap methyltransferase 1 ; ELAVL1, ELAV like RNA binding protein 1; PLAUR, plasminogen activator, urokinase receptor; GAPDH, glyceraldehyde-3-phosphate dehydrogenase.

Table S2 Primer sets used for constructs

Primer set	Primers	Sequence
CV186-SNORA37	Forward	5'-CGCGGATCCTGAGCACTTTCACAGGTCCTCC-3'
	Reverse	5'-GCGCACCGGTAATTGTCCCATTGAATGACAGCT-3'
CV186-CMTR1	Forward	5'-CAGGTCGACTCTAGAGGATCCATGAAGAGGAGAACTGACCC-3'
	Reverse	5'-ATCCTTGTAGTCCATACCGGTTTAGGCCCTGTGCATCTGGA-3'
pCMV-HA-CMTR1	Forward	5'-ACGCGTCGACGATGAAGAGGAGAACTGACCC-3'
	Reverse	5'-ATTTGCGGCCGCTTAGGCCCTGTGCATCTGGA-3'
pCMV-HA-CMTR1-ΔNLS	Forward	5'-ACGCGTCGACGTCCATGTATAATAGCGTCTC-3'
	Reverse	5'-ATTTGCGGCCGCTTAGGCCCTGTGCATCTGGA-3'
pCMV-HA-CMTR1-ΔNLS-G-patch	Forward	5'-ACGCGTCGACGGAAATGAGCGATTGGATGGT-3'
	Reverse	5'-ATTTGCGGCCGCTTAGGCCCTGTGCATCTGGA-3'
pCMV-HA-CMTR1-ΔNLS-G-patch-WW	Forward	5'-ACGCGTCGACGGAAATGAGCGATTGGATGGT-3'
	Reverse	5'-ATTTGCGGCCGCATTCACTGTCCTGACGATGT-3'
pGEX-6p-1-CMTR1	Forward	5'-ACGCGTCGACGGATGAAGAGGAGAACTGACCC-3'
	Reverse	5'-ATTTGCGGCCGCTTAGGCCCTGTGCATCTGGA-3'
pGEX-6p-1-CMTR1-ΔNLS	Forward	5'-ACGCGTCGACGGTCCATGTATAATAGCGTCTC-3'
	Reverse	5'-ATTTGCGGCCGCTTAGGCCCTGTGCATCTGGA-3'
pGEX-6p-1-CMTR1-ΔNLS-G-patch	Forward	5'-ACGCGTCGACGGGAAATGAGCGATTGGATGGT-3'
	Reverse	5'-ATTTGCGGCCGCTTAGGCCCTGTGCATCTGGA-3'
pGEX-6p-1-CMTR1-ΔNLS-G-patch-WW	Forward	5'-ACGCGTCGACGGGAAATGAGCGATTGGATGGT-3'
	Reverse	5'-ATTTGCGGCCGCATTCACTGTCCTGACGATGT-3'
pMAL-c4X-ELAVL1	Forward	5'-CCGGAATTCATGTCTAATGGTTATGAAGACC-3'
	Reverse	5'-ACGCGTCGACTTATTTGTGGGACTTGTTGGTT-3'
pMAL-c4X-ELAVL1-ΔRRM3	Forward	5'-CCGGAATTCATGTCTAATGGTTATGAAGACC-3'
	Reverse	5'-ACGCGTCGACCCAGCCGGAGGAGGCGTTT-3'
pMAL-c4X-ELAVL1-RRM2+H	Forward	5'-CCGGAATTCATCAAAGACGCCAACTTGTACA-3'
	Reverse	5'-ACGCGTCGACCCAGCCGGAGGAGGCGTTT-3'
pMAL-c4X-ELAVL1-RRM2	Forward	5'-CCGGAATTCATCAAAGACGCCAACTTGTACA-3'
	Reverse	5'-ACGCGTCGACCTGGTTGGGGTTGGCTGCA-3'
pBiFC-CMTR1-VN173	Forward	5'-CGCGCGGCCGCGATGAAGAGGAGAACTGACCC-3'
	Reverse	5'-CGCGGTACCGCGGCCCTGTGCATCTGGATGA-3'
pBiFC-ELAVL1-VC155	Forward	5'-CCGGAATTCGGATGTCTAATGGTTATGAAGA-3'
	Reverse	5'-CCGCTCGAGTTTGTGGGACTTGTTGGTTT-3'

SNORA37, small nucleolar RNA, H/ACA box 37; CMTR1, cap methyltransferase 1; ELAVL1, ELAV like RNA binding protein 1.

Table S3 Oligonucleotide sets for short hairpin RNAs

Primer set	Sequence
sh-Scb	5'-AGGGATACAAGCATATACCACTCGAGTGGTATATGCTTGTATCCCTC-3' (sense);
	5'-GAGGGATACAAGCATATACCACTCGAGTGGTATATGCTTGTATCCCT-3' (antisense)
sh-SNORA37 #1	5’-CCGGTCTGTCATTCAATGGGACAATTCTCGAGAATTGTCCCATTGAATGACAGTTTTTG-3’ (sense)
	5’-GATCCAAAAACTGTCATTCAATGGGACAATTCTCGAGAATTGTCCCATTGAATGACAGA-3’ (antisense)
sh-SNORA37 #2	5’-CCGGTGGTAGCTGGTGCTGAGAGAAACTCGAGTTTCTCTCAGCACCAGCTACCTTTTTG-3’ (sense)
	5’-GATCCAAAAAGGTAGCTGGTGCTGAGAGAAACTCGAGTTTCTCTCAGCACCAGCTACCA-3’ (antisense)
sh-CMTR1 #1	5’-CCGGTGCATGCAAAGGGCTTTGGAATCTCGAGATTCCAAAGCCCTTTGCATGCTTTTTG-3’ (sense)
	5’-GATCCAAAAAGCATGCAAAGGGCTTTGGAATCTCGAGATTCCAAAGCCCTTTGCATGCA-3’ (antisense)
sh-CMTR1 #2	5’-CCGGTGCATAGATGATGTTCGGGATTCTCGAGAATCCCGAACATCATCTATGCTTTTTG-3’ (sense)
	5’-GATCCAAAAAGCATAGATGATGTTCGGGATTCTCGAGAATCCCGAACATCATCTATGCA-3’ (antisense)
sh-ELAVL1 #1	5'-CCGGACCATGACAAACTATGAAGAACTCGAGTTCTTCATAGTTTGTCATGGTTTTTTG -3' (sense);
	5'-GATCCAAAAAACCATGACAAACTATGAAGAACTCGAGTTCTTCATAGTTTGTCATGGT-3' (antisense)
sh-ELAVL1 #2	5'-CCGGCCCATCACAGTGAAGTTTGCACTCGAGTGCAAACTTCACTGTGATGGGTTTTTG-3' (sense);
	5'-GATCCAAAAACCCATCACAGTGAAGTTTGCACTCGAGTGCAAACTTCACTGTGATGGG-3' (antisense)

SNORA37, small nucleolar RNA, H/ACA box 37; CMTR1, cap methyltransferase 1; ELAVL1, ELAV like RNA binding protein 1.

Table S4 Differentially expressed snoRNAs in RNA-seq assay

Gene ID	*P*-Value	logFC
SNORD105B	2.02E-02	3.814643318
SNORD116-26	2.45E-02	3.730589199
SNORA37	1.77E-02	2.169396966
SNORD110	3.52E-02	1.367205527
SNORD68	4.60E-02	1.171923731
SNORD87	2.30E-02	1.165189333
SNORD33	2.66E-02	1.142674046
SNORD32A	4.65E-02	1.03081066
SNORD91B	4.47E-02	-1.090525745
SNORD58	3.18E-02	-1.144579757
SNORD28	2.04E-02	-1.677140888
SNORD116-25	3.72E-02	-2.137392071
SNORA28	2.38E-02	-2.474951099
SNORA42	1.80E-02	-2.764101686

Table S5 Alteration of splicing events in MKN-45 cells upon *SNORA37* over-expression

Gene (top 60)	AS type	Chromosome	*P*-value	△PSI
AAK1	SE	chr2	4.31E-14	0.878
ABI3BP	SE	chr3	2.74E-06	0.583
ANAPC15	A5SS	chr11	1.44E-08	0.874
ANXA7	SE	chr10	9.23E-03	0.534
CCDC78	MXE	chr16	1.33E-05	-0.597
CCM2	SE	chr7	5.11E-13	-0.796
CD44	SE	chr12	4.78E-07	0.667
COA1	SE	chr7	8.30E-04	0.537
COL16A1	SE	chr1	2.15E-03	-0.556
CTDSPL	SE	chr3	9.07E-06	-0.667
DTX2	SE	chr7	2.88E-07	-0.556
EEF1D	SE	chr8	1.39E-04	0.598
EPHA10	SE	chr1	2.71E-02	0.545
FAM49B	MXE	chr8	2.49E-04	-0.552
FANCL	SE	chr2	3.60E-06	-0.582
FXR1	SE	chr3	9.17E-05	-0.577
GDPD5	SE	chr11	1.36E-07	0.743
GRB10	SE	chr7	1.31E-04	0.535
HACL1	SE	chr3	1.68E-08	-0.872
HARS2	SE	chr5	3.83E-05	-0.569
IFI27L1	SE	chr14	5.82E-08	-0.729
IL20RA	MXE	chr6	8.42E-05	-0.621
KLHL3	SE	chr5	3.50E-05	0.599
KLK8	SE	chr19	1.09E-08	0.7
LRRC23	SE	chr12	8.35E-05	0.576
METAP2	SE	chr12	5.33E-04	0.57
MGME1	A5SS	chr20	3.63E-03	-0.58
MTHFSD	SE	chr16	3.47E-08	0.852
MTIF3	SE	chr13	1.21E-08	0.723
NDUFC1	SE	chr4	2.65E-06	0.604
NPRL3	SE	chr16	2.85E-03	0.727
NQO2	SE	chr6	5.74E-09	-0.543
NSMCE2	SE	chr8	9.23E-03	-0.667
PILRB	A5SS	chr7	1.43E-05	-0.528
PNPLA6	A5SS	chr19	3.02E-04	-0.529
POU5F1	RI	chr6	2.48E-06	0.609
PPP1R3E	SE	chr14	8.04E-06	0.562
PPRC1	SE	chr10	5.01E-08	-0.617
PRMT2	SE	chr8	1.39E-04	-0.742
PSME2	A5SS	chr14	2.85E-04	0.517
PXMP4	SE	chr20	7.00E-05	0.545
RACGAP1	SE	chr12	1.09E-03	0.555
RAD51D	SE	chr17	1.42E-06	-0.603
RBPMS	SE	chr8	1.33E-02	0.518
RPGRIP1L	SE	chr16	9.74E-07	-0.664
RRP8	SE	chr11	6.44E-03	-0.591
RTN3	SE	chr11	7.17E-04	-0.667
SLC37A4	A5SS	chr11	1.75E-06	-0.609
SLC6A9	SE	chr1	3.03E-03	-0.585
SLCO2A1	SE	chr3	2.23E-03	0.558
SMTN	SE	chr22	3.30E-03	-0.562
SPC24	SE	chr19	6.91E-07	0.51
SPINK5	SE	chr5	2.98E-06	0.609
STK33	SE	chr11	1.17E-05	-0.607
TMEM161B-AS1	SE	chr5	3.89E-07	0.618
TRPV2	SE	chr17	4.64E-02	-0.539
TSGA10	SE	chr2	4.96E-10	0.832
WBSCR22	SE	chr7	3.68E-06	-0.54
ZBTB8OS	SE	chr1	1.22E-02	-0.529
ZFYVE27	SE	chr10	2.94E-02	0.545

AS, alteration splicing; SE, skipping exon; A5SS, alternative 5′ splice sites; MXE, mutually exclusive exons.

Table S6 Alteration of splicing events in gastric tissues

Gene (top 60)	AS type	Chromosome	*P* value	△PSI
AC004076.9	SE	chr19	1.75E-06	0.656
AC034220.3	MXE	chr5	4.46E-08	-0.612
ARPC1B	A3SS	chr7	4.01E-06	0.845
BDH1	SE	chr3	9.34E-07	0.753
BORA	SE	chr13	2.58E-08	-0.605
CACNA2D1	A3SS	chr7	5.66E-12	-0.721
CCDC18-AS1	SE	chr1	8.60E-07	0.602
CCDC81	SE	chr11	2.66E-02	-0.667
CD44	SE	chr21	9.28E-04	0.585
CEP295	A3SS	chr11	1.29E-04	0.61
CPSF3L	SE	chr1	1.90E-04	-0.746
DOCK9	MXE	chr13	1.73E-03	0.779
DST	SE	chr6	1.47E-07	0.616
DTNB	SE	chr2	1.94E-05	-0.649
ELMOD3	SE	chr2	6.38E-06	0.613
ENC1	SE	chr5	1.45E-12	0.67
EP400NL	SE	chr12	1.59E-06	0.695
ESRP1	SE	chr8	4.41E-05	0.622
EXO5	SE	chr1	6.32E-08	-0.667
FLT3LG	A3SS	chr19	3.14E-08	-0.74
GTPBP3	A3SS	chr19	5.49E-04	-0.637
HKR1	SE	chr19	1.56E-03	-0.726
LINC00893	SE	chrX	5.65E-03	0.614
LRRC37A2	SE	chr17	9.97E-05	-0.823
MAPK8	SE	chr10	1.74E-10	-0.602
METTL21B	SE	chr12	9.32E-06	-0.732
MOK	A3SS	chr14	4.23E-05	-0.585
NCOA3	A5SS	chr20	9.84E-11	0.729
NDE1	SE	chr16	6.22E-07	-0.667
NUDT13	SE	chr10	1.85E-03	0.653
PRMT2	SE	chr5	2.38E-06	-0.696
PSEN1	SE	chr14	5.68E-07	-0.662
PUM2	SE	chr2	1.60E-08	-0.688
RFWD2	SE	chr1	1.06E-03	0.593
SEMA4C	A3SS	chr2	7.60E-05	0.634
SIGLEC11	MXE	chr19	3.07E-12	0.883
SIGLEC11	SE	chr19	4.88E-07	-0.747
SLC12A8	SE	chr3	2.09E-07	-0.687
SLC25A19	SE	chr17	1.10E-07	0.736
SLC43A3	SE	chr11	4.14E-03	0.583
SPET--4	SE	chr2	3.99E-08	0.805
ST7L	MXE	chr1	6.21E-07	0.583
TBC1D5	SE	chr3	1.10E-07	-0.801
TCF3	RI	chr19	5.40E-03	-0.637
TEX9	SE	chr15	1.96E-02	-0.618
TRIM6	SE	chr11	5.22E-06	-0.695
TRIQK	SE	chr8	1.03E-03	0.665
TTC23	SE	chr15	6.16E-06	-0.609
TTC8	SE	chr14	6.77E-08	0.602
ULK4	MXE	chr3	5.45E-11	0.679
UPP1	SE	chr7	1.66E-05	0.667
ZNF226	RI	chr19	6.68E-09	0.809
ZNF3	SE	chr7	7.61E-11	-0.652
ZNF346	MXE	chr5	4.29E-08	0.784
ZNF35	A5SS	chr3	1.62E-07	0.684
ZNF415	MXE	chr19	1.66E-06	-0.595
ZNF551	SE	chr19	3.83E-07	-0.597
ZNF585B	SE	chr19	3.12E-07	-0.586
ZNF611	SE	chr19	5.65E-08	0.585
ZNF7	A3SS	chr8	9.08E-07	-0.616

AS, alteration splicing; SE, skipping exon; MXE, mutually exclusive exons; A3SS, alternative 3′ splice sites; A5SS, alternative 5′ splice sites.

Table S7 Mass spectrometry analysis of proteins pulled down by *SNORA37*

ADPRS	GOLGB1	POLR1B	ZNF106
AKAP17A	GTPBP1	POLR1E	ZNF668
APOE	IFIT3	POLR3G	ZRANB3
BAG3	IL13	PSMD8	ZSWIM5
BNC2	ISG20L2	RAP2B	
BRPF3	KCMF1	RBFOX1	
CBX2	KCNJ10	RFC5	
CEP250	KRIT1	RHBDD2	
CISD2	LGALSL	RPA1	
CLASP1	LRRC14	SDF2L1	
CMTR1	MDN1	SEC24A	
COMMD4	MED9	SNX29	
CRK	MLH1	SP110	
CRYZ	MLLT3	SPAST	
CSN1S1	MRE11	SPDL1	
CSNK1E	MROH2A	ST3GAL4	
DAD1	MRPS28	ST6GALNAC5	
DCAKD	MSI2	TBC1D15	
DDX19A	MSX2	TFB1M	
DEFB113	MX1	TFIP11	
DIAPH3	MYRF	TMEM97	
DYTN	NDUFS4	TOLLIP	
ENSA	NT5C3A	TRIM61	
EPN3	NVL	UGT8	
ERI1	PAK4	UTP4	
ESF1	PCOLCE	VKORC1L1	
FKBP8	PDP1	XRCC4	
FNDC3B	PI3	YES1	
FOCAD	PICALM	ZCCHC17	
GNG12	PNPLA2	ZFC3H1	
